# Morphology of the bryozoan *Cinctipora elegans* (Cyclostomata, Cinctiporidae) with first data on its sexual reproduction and the cyclostome neuro-muscular system

**DOI:** 10.1186/s12862-018-1206-1

**Published:** 2018-06-14

**Authors:** Thomas F. Schwaha, Stephan Handschuh, Andrew N. Ostrovsky, Andreas Wanninger

**Affiliations:** 1 Faculty of Life Sciences, Department of Integrative Zoology, University of Vienna, Althanstraße 14, 1090 Vienna, Austria; 2 VetCore Facility for Research, University of Veterinary Medicine, Veterinärplatz 1, 1210 Vienna, Austria; 3 Faculty of Earth Sciences, Geography and Astronomy, Department of Palaeontology, University of Vienna, Geozentrum, Althanstraße 14, 1090 Vienna, Austria; 4 Faculty of Biology, Department of Invertebrate Zoology, Saint Petersburg State University, Universitetskaja nab. 7/9, 199034 Saint Petersburg, Russia

**Keywords:** *C. elegans*, Myoanatomy, Nervous system, Serotonin, Tubulin, Lophotrochozoa, Evolution, Morphology

## Abstract

**Background:**

Cyclostome bryozoans are an ancient group of marine colonial suspension-feeders comprising approximately 700 extant species. Previous morphological studies are mainly restricted to skeletal characters whereas data on soft tissues obtained by state-of-the-art methods are still lacking. In order to contribute to issues related to cyclostome ground pattern reconstruction, we analyzed the morphology of the neuromuscular system *Cinctipora elegans* by means of immunocytochemical staining, confocal laser scanning microscopy, histological sections and microCT imaging.

**Results:**

Polypides of *C. elegans* are located in elongated tubular skeletal cystids. Distally, the orifice leads into a prominent vestibulum which is lined by an epithelium that joins an almost complete perimetrical attachment organ, both containing radially arranged neurite bundles and muscles. Centrally, the prominent atrial sphincter separates the vestibulum from the atrium. The latter is enclosed by the tentacle sheath which contains few longitudinal muscle fibers and two principal neurite bundles. These emerge from the cerebral ganglion, which is located at the lophophoral base. Lateral ganglia are located next to the cerebral ganglion from which the visceral neurite bundles emerge that extend proximally towards the foregut. There are four tentacle neurite bundles that emerge from the ganglia and the circum-oral nerve ring, which encompasses the pharynx. The tentacles possess two striated longitudinal muscles. Short buccal dilatators are situated at the lophophoral base and short muscular sets are present at the abfrontal and frontal side of the tentacle base. The pharynx is myoepithelial and triradiate in cross-section. Oocytes are found inside the pharyngeal myoepithelium. The digestive tract contains dense circular musculature and few longitudinal muscles. The membranous sac contains regular, thin, circular and diagonal muscles and neurites in its epithelial lining.

**Conclusions:**

The general structure of the neuro-muscular system is more reminiscent of the condition found in Gymnolaemata rather than Phylactolaemata, which supports a close relationship between Cyclostomata and Gymnolaemata. Several characters of *C. elegans* such as the lateral ganglia or loss of the cardia are probably apomorphic for this species. For the first time, oocytes that surprisingly develop in the pharyngeal wall are reported for this species.

## Background

Bryozoa is a phylum of aquatic colonial suspension feeders comprising over 6.000 recent and 15.000–20.000 fossil species [1]. The phylum is known since the lower Ordovician and is widely distributed from the intertidal to the abyss and, together with sponges and cnidarians, dominates various benthic communities [2, 3]. Such historical longevity and evolutionary success measured in terms of taxonomic diversity is explained by very high adaptive potential and evolutionary plasticity of this group manifested on various levels of zooidal and colonial organization [2, 3].

Bryozoan colonies consist of iterative modules (zooids) whose relative position and budding patterns result in the great diversity of colonial growth forms adapted to various habitats [4]. Each zooid consists of a polypide comprising the ciliated tentacle crown (the lophophore), a u-shaped digestive tract and associated muscular and neural tissue, and the cystid, which consists of the protective body wall and its cuticle as well as any mineralized skeleton [5]. Typical for bryozoans is the retraction process where the polypide is withdrawn into the protective cystid via prominent retractor muscles.

Three major taxa are commonly recognized: Phylactolaemata, Stenolaemata and Gymnolaemata. Phylactolaemata only occurs in freshwater whereas the other two clades comprise chiefly marine animals. Phylactolaemata is considered the sister group to the remaining clades with Stenolaemata being sister to Gymnolaemata [6, 7]. Cyclostome bryozoans are a group of approximately 700 species that represent the only extant taxon of the once more diverse Stenolaemata. The earliest fossils occur in the lower Ordovician where this small clade coexisted alongside the much more speciose stenolaemates that became extinct near the Paleozoic-Mesozoic boundary. Cyclostomes survived, however, and underwent an explosive radiation and became the dominant bryozoan group from the Jurassic until the mid Late Cretaceous [8–11]. Despite their strong decline after the Cretaceous-Tertiary boundary, they are still abundant and constitute up to 20% of bryozoan faunas [2, 12].

One of the key features of all stenolaemates is calcification of the cystid walls – a character shared with the gymnolaemate cheilostomes, but which evolved independently [13, 14]. Apomorphies of the post-Palaeozoic Cyclostomata are gonozooids, voluminous polymorphic zooids for larval incubation, and polyembryony that gives rise to clonal larvae [15–17]. Another unique character, probably for all stenolaemates, is the so-called membranous sac. Retraction in all bryozoans is facilitated via retractor muscles, but the protrusion mechanisms differ among the various clades. Due to their calcified cystids, the body wall is not deformable, thus calling for a special protrusion mechanism in cyclostomes (and probably all stenolaemates). In this group, the peritoneal layer is detached from the body wall to form the membranous sac [15]. Annular ring muscles in the linings of this sac allow pressure changes in the contained coelomic cavity, thereby causing polypide protrusion [18].

**Fig. 1:**
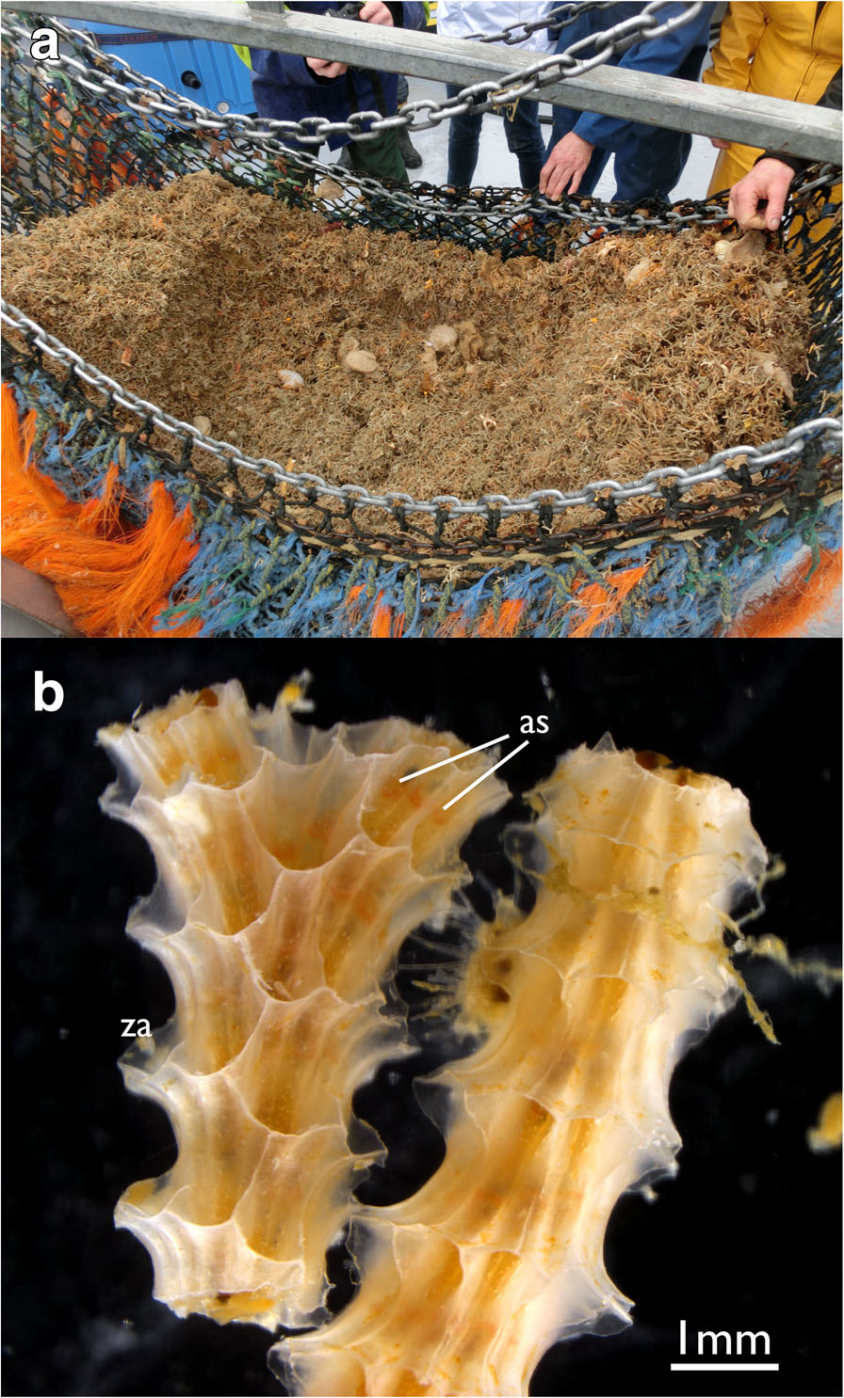
*Cinctipora elegans*. **a** Image showing its abundance in certain areas. **b** Two colony fragments (right fragment upside down) showing the arrangement of zooids. The attachment sites of polypides to the skeleton in the apertural area are visible as orange hue. Abbreviations: as – attachment sites of the polypides in the apertural area. za – zooidal aperture

Cinctiporidae is an cyclostome family known since the upper Cretaceous. The earliest representative is *Cinctipora* sp. from the Maastrichtian [19]. The only recent forms are *Cinctipora elegans* and *Attinopora zealandica* from New Zealand [20], where *C. elegans* locally is particularly abundant (Fig.1a). Cinctiporids are unique among post-Palaeozoic cyclostomes by possessing untypically large zooids, while characteristic incubation chambers (gonozooids), otherwise diagnostic for recent cyclostomes, are missing. Virtually nothing is known about their sexual reproduction except for morphological descriptions of their ancestrulae (founding zooids by settlement of a larva) [20]. Another peculiar feature is that zooids of *C. elegans* possess both, either an external or internal calcified skeleton within a colony (fixed-walled or free-walled) [20, 21]. Some of these unique features have been used to argue for an early branching of this family within Cyclostomata. However, the first molecular-based phylogenies failed to support this hypothesis [22] and its position is currently inconclusively resolved [6].

Cinctiporid skeletal morphology has been previously described in a large monograph which specifically focused on colony architecture and formation as well as zooidal wall morphology. Polypide anatomy was also studied using histological sections [20]. In general, however, information on soft body morphology of cyclostomes is extremely scarce [23] with most of our knowledge dating back to the work of Borg [15] which covered morphological descriptions and anatomy of about 40 species from nine families. Few additional studies were mainly focused on specific organ systems such as tentacles or the digestive tract [18, 24–27]. Still, our knowledge is very fragmentary and almost nothing is known on the cyclostome neuro-muscular systems.

In the past years, several studies have focused on bryozoan neuro-muscular systems using fluorescence labeling and confocal laser scanning microscopy (CLSM) [28–30]. However, so far, only phylactolaemate [29, 31] and gymnolaemate [30, 32, 33] representatives were studied; data on cyclostomes are still lacking.

Since cyclostomes represent the most basally branching skeletal bryozoans and constitute the sister-group to the gymnolaemates, their study is vital for our understanding of the morphological evolution of characters within this large group of colonial suspension feeders. In order to fill this gap in knowledge we studied the cyclostome *C. elegans* with fluorescence stains of neuro-muscular components and microCT supplemented by histological sectioning techniques.

## Methods

### Sampling

Samples were collected in October 2016 by dredging at various localities close to South Island, New Zealand, in the vicinity of Stewart Island. Samples were either fixed for sectioning techniques in 2.5% glutaraldehyde in 0. 1 M sodium-cacodylate buffer (ph 7.4) for several days (~ 5) at 4 °C, or for fluorescence staining in 4% paraformaldehyde in 0.1 M phosphate buffer (PB; ph 7.4) for one hour. Afterwards, samples were rinsed several times in PB and further processed or stored until further preparation in PB with ~ 0.1% NaN_3_ added. Samples for microCT were fixed and stored in 70% ethanol.

### Sectioning

For sectioning, samples were post-fixed in ~ 1% aqueous osmium tetroxide for one hour at room temperature followed by several rinses in distilled water. Afterwards, samples were decalcified in 2–4% ascorbic acid for two to three weeks with daily changes. Dehydration was conducted with acidified 2–2-dimethoxypropane and acetone was used as intermediate to transfer dehydrated specimens into Agar LVR resin (Agar Scientific, Stansted, Essex, UK). Ribbons of serial semithin sections of 1 μm thickness were conducted with a Diatome HistoJumbo diamond knife (Diatome, Biel, Switzerland) on a Leica UC6 ultramicrotome (Leica Microsytems, Wetzlar, Germany) as described by Ruthensteiner [34]. Staining was performed with toluidine blue for a few seconds at 60 °C. Sections were photographed with a Nikon Eclipse E800 with a Nikon DsFi3-U3 microscope camera or a Nikon NiU with a DsRi2 microscope camera (Nikon, Tokyo, Japan).

### Fluorescence staining

Prior to staining, samples were decalcified as mentioned above or with 0.05 M EGTA. Decalcified samples were incubated in 6% normal goat serum (NGS) in 2% Triton-X 100 and 2% dimethylsulfoxide in phosphate buffer (pH 7.4) (PTD) for approximately 24 h at room temperature. Primary antibody combinations against acetylated alpha-tubulin from mouse (Sigma Aldrich, St. Louis, MO) and rabbit antibodies against serotonin (Immunostar, Hudson, USA) were applied at a concentration of 1:1000 each diluted in PTD for ~ 24 h at room temperature. This was followed by several washes with PB for about two hours followed by incubation with fluorochrome-conjugated secondary antibodies raised in goat against either mouse (AlexaFluor568, Invitrogen, Carlsbad, CA) or rabbit (AlexaFluor633, Invitrogen) at a concentration of 1:300 diluted in PTD. For f-actin visualization Bodipy FL phallacidin (Molecular Probes, Eugene, OR, USA) was applied at a concentration of ~ 1:40 and DAPI was added at a concentration of 1:600 for nucleic acid staining together with the secondary antibodies. Incubation lasted ~ 24 h at room temperature. Afterwards, samples were washed in PB for three or four times before being mounted in Fluoromount G (Southern Biotech, Birmingham, AL) on glass slides. Confocal image stacks were recorded with a Leica SP5 II confocal laser scanning microscope (Leica). Data analysis and volume renderings were performed with Amira 6.1.1 (FEI Visualization Sciences Group, Mérignac Cédex, France) or FIJI [35].

### MicroCT

MicroCT scans were acquired for the purpose of simultaneously visualizing the mineralized colony skeleton and soft tissues of zooids. Colonies were cut into pieces of 5–10 mm length. To enhance soft tissue contrast, one specimen was stained with 1% phosphotungstic acid (PTA) in 70% ethanol for 48 h [36]. After staining, the sample was rinsed in 70% ethanol for 24 h to remove unbound PTA from tissues. For scanning, the specimen was vertically mounted in 1.5% low melt Agarose in a heat-sealed plastic pipette tip. MicroCT scans were acquired using a Zeiss XRadia microXCT-400 (Carl Zeiss X-ray microscopy, Pleasanton, CA). Source peak voltage was 40kVp and intensity was 100 μA. First, an overview tomography scan was acquired showing the whole colony diameter (FOV = 2.36 mm) using the 10× optical magnification lens with 70 mm source-to-sample distance and 10 mm detector-to-sample distance, an exposure time of 60 s per projection, and an angular increment of 0.2° between projections. Isotropic voxel resolution in the reconstructed volume was 2.34 μm. Second, a high resolution interior tomography scan from several zooids (FOV = 1.18 mm) was acquired using the 20× optical magnification lens with 70 mm source-to-sample distance and 10 mm detector-to-sample distance, an exposure time of 100 s per projection, and an angular increment of 0.1667° between projections. Isotropic voxel resolution in the reconstructed volume was 1.19 μm. To cover the whole length of elongated zooids, two interior tomographies were acquired at a vertical offset of 0.8 mm and stitched with the Plugin_Stich.dll plugin of the XM Controller software.

Reconstructed volumes were saved as *.txm files and imported into the 3D reconstruction software Amira 6.3 (FEI Visualization Sciences Group, Mérignac Cédex, France). The mineralized colony skeleton was segmented semi-automatically using intensity based segmentation tool in the Amira Segmentation editor. Subsequently, the mineralized colony skeleton was saved as a separate image volume. Soft tissues of zooids were also saved as a separate image volume by subtraction of the colony skeleton from the original image volume. Colony skeleton and soft tissue image volumes were visualized using the volume rendering software Drishti [37].

## Results

### Gross morphology

**Fig. 2:**
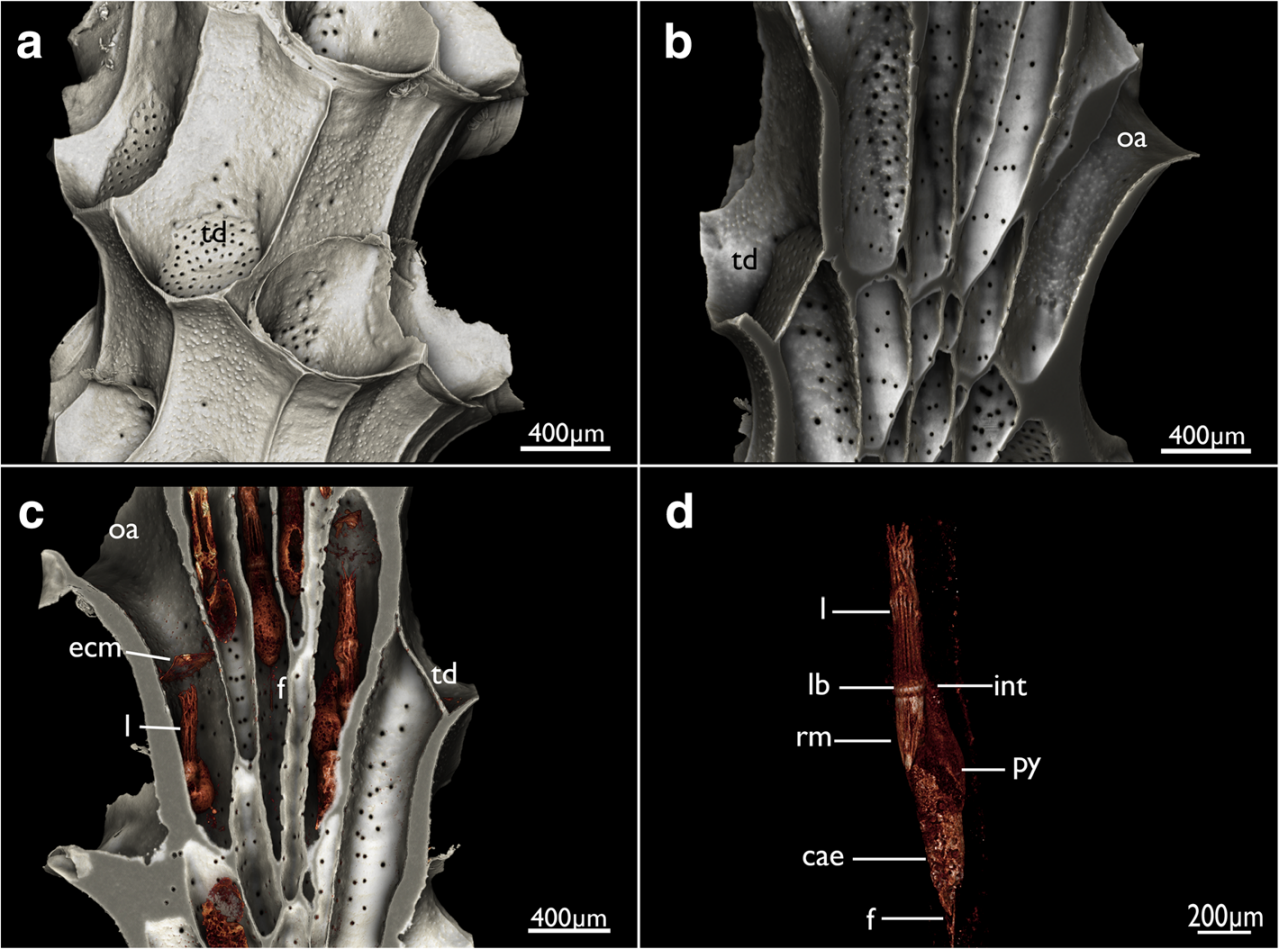
Overview of *Cinctipora elegans* from microCT images of the skeletal parts (white) and the soft-body party stained with phosphotungstic acid (reddish-brown). **a** Volume rendering of the skeleton of a part of a colony showing the mineralized skeleton of several zooids. Several interzooidal communication pores are evident on the skeletal walls. Some apertures are closed by calcareous diaphragms. **b** Longitudinal section through a volume rendering of the mineralized skeleton showing zooidal arrangement and open vs. closed apertures. **c** Longitudinal section through a volume rendering of the skeleton including polypides showing the relative position of the polypides within the skeleton. This rendering shows that the attachment organ typically lies more than 500 μm proximally of the open skeletal aperture. **d** Volume rendering of the polypide of a single zooid. Abbreviations: cae – caecum, ecm – thick extracellular matrix of the attachment organ, f – funiculus, int – intestine, l – lophophore, lb – lophophoral base, oa – open aperture, py – pylorus, rm – retractor muscle, td – terminal diaphragm

**Fig. 3:**
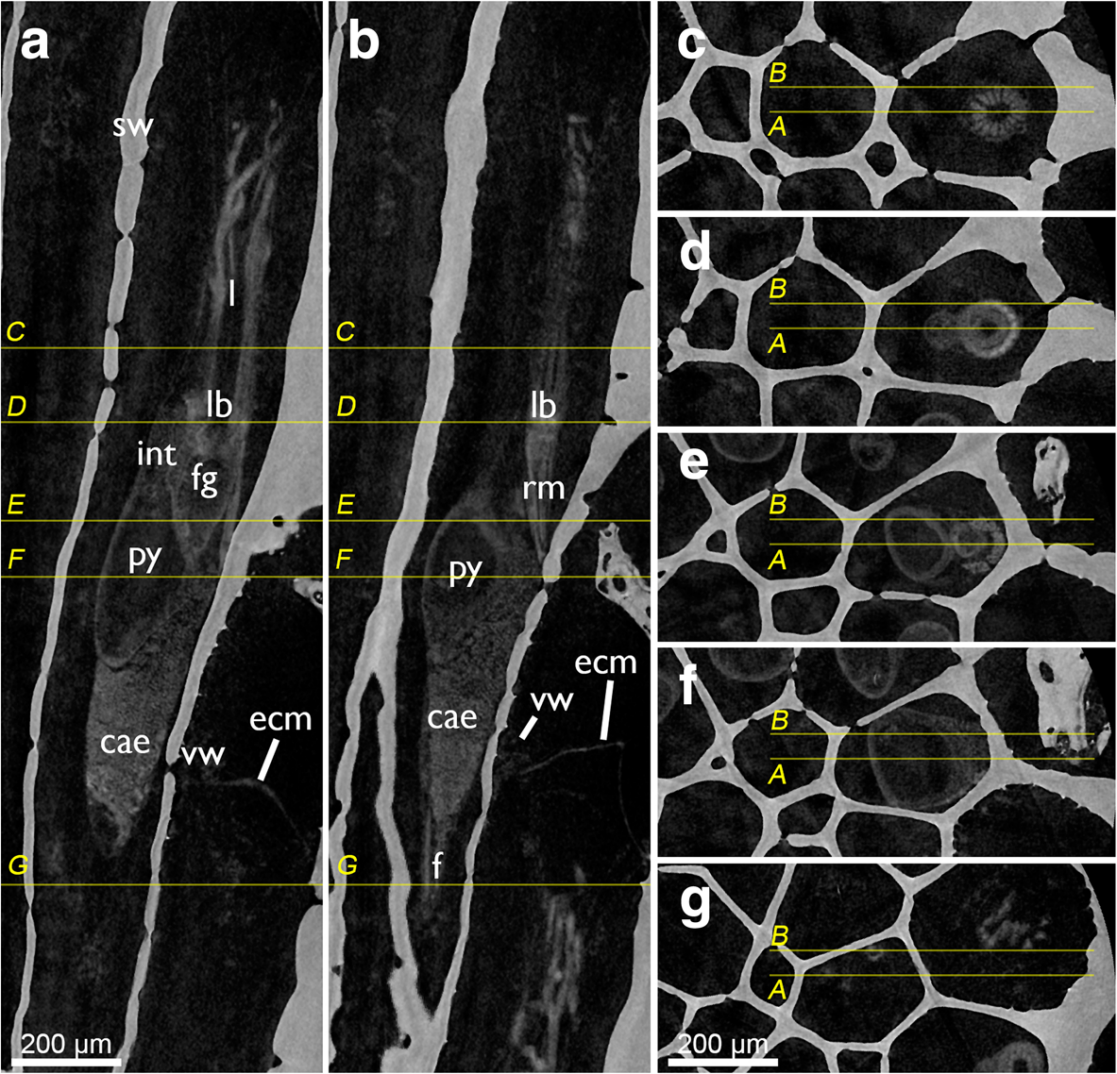
Tomographic sections of skeleton and soft-body visualization by microCT of *Cinctipora elegans*. **a** and **b** are longitudinal sections of two zooids showing the homogenous skeletal wall and the lighter shades of grey of the polypide. The yellow lines indicate cross-sectional planes displayed in **c**-**g** which progress from distal to proximal. Abbreviations: cae – caecum, ecm – thick extracellular matrix of the attachment organ, f – funiculus, fg – foregut, int – intestine, l – lophophore, lb. – lophophoral base, py – pylorus, rm. – retractor muscle, sw – skeletal wall, vm – vestibular wall

**Fig. 4:**
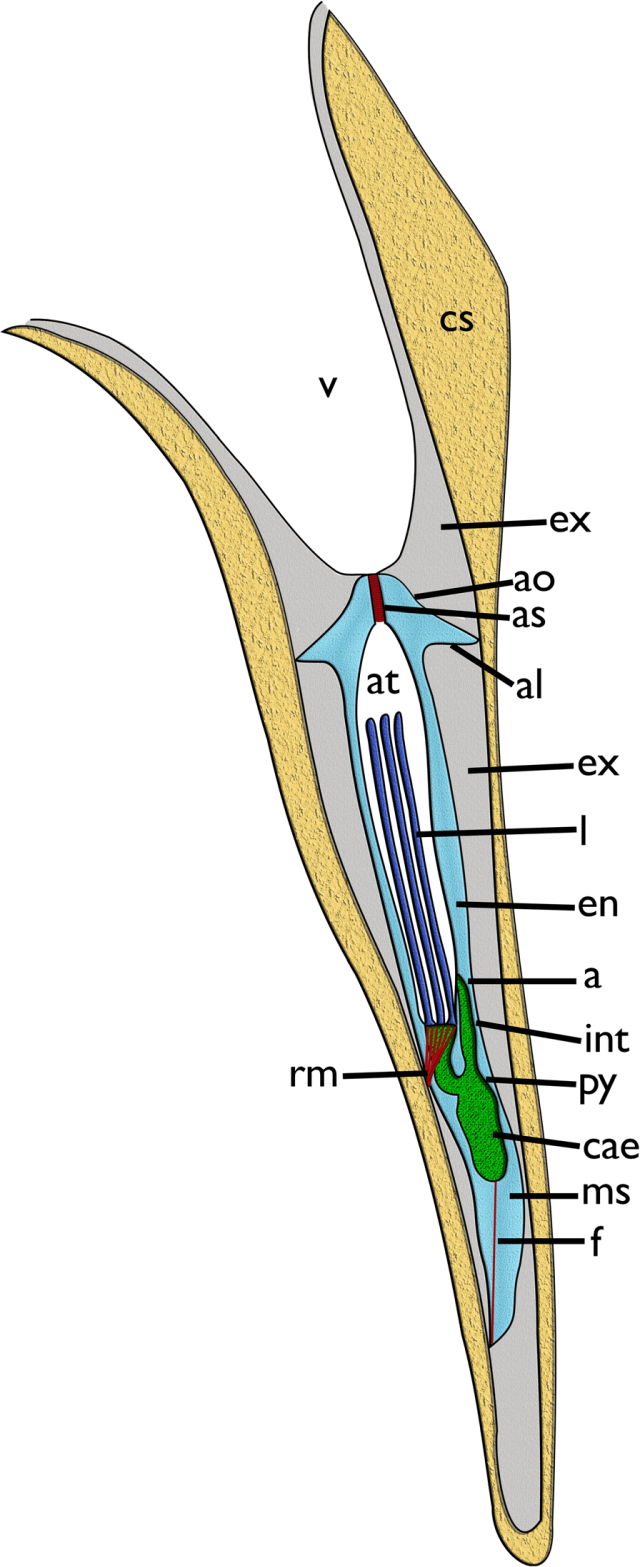
Schematic longitudinal section of a single zooid of *Cinctipora elegans* with the skeletal parts of the cystid forming a tube around the polypide. The calcified parts of the skeleton are displayed in light brown. The orifice leads into the vestibulum that continues via the atrial sphincter into the atrium. The latter is formed by the inverted tentacle sheath around the retracted lophophore (dark blue). At the lophophoral base lies the mouth opening. The gut (green) is u-shaped with a short foregut leading into a voluminous caecum and further via a pylorus and intestine into the anus that opens into the tentacle sheath. Cyclostomes have a membranous sac which constitutes the peritoneum detached from the epidermis to form an exosaccal cavity (primary body cavity) and endosaccal cavity (coelom or secondary body cavity, light blue). Prominent retractor muscles origin from the body wall and insert at the lophophoral base. A muscular funiculus connects the caecum to the proximal area of the membranous sac. Abbreviations: a – anus, al – attachment ligament, ao – attachment organ, as – atrial sphincter, at – atrium, cae – caecum, cs – calcified skeleton, en – endosaccal space (coelom), ex – exosaccal space, f – funiculus, int – intestine, l – lophophore, ms – membranous sac, py – pylorus, rm – retractor muscle, v – vestibulum

**Fig. 5:**
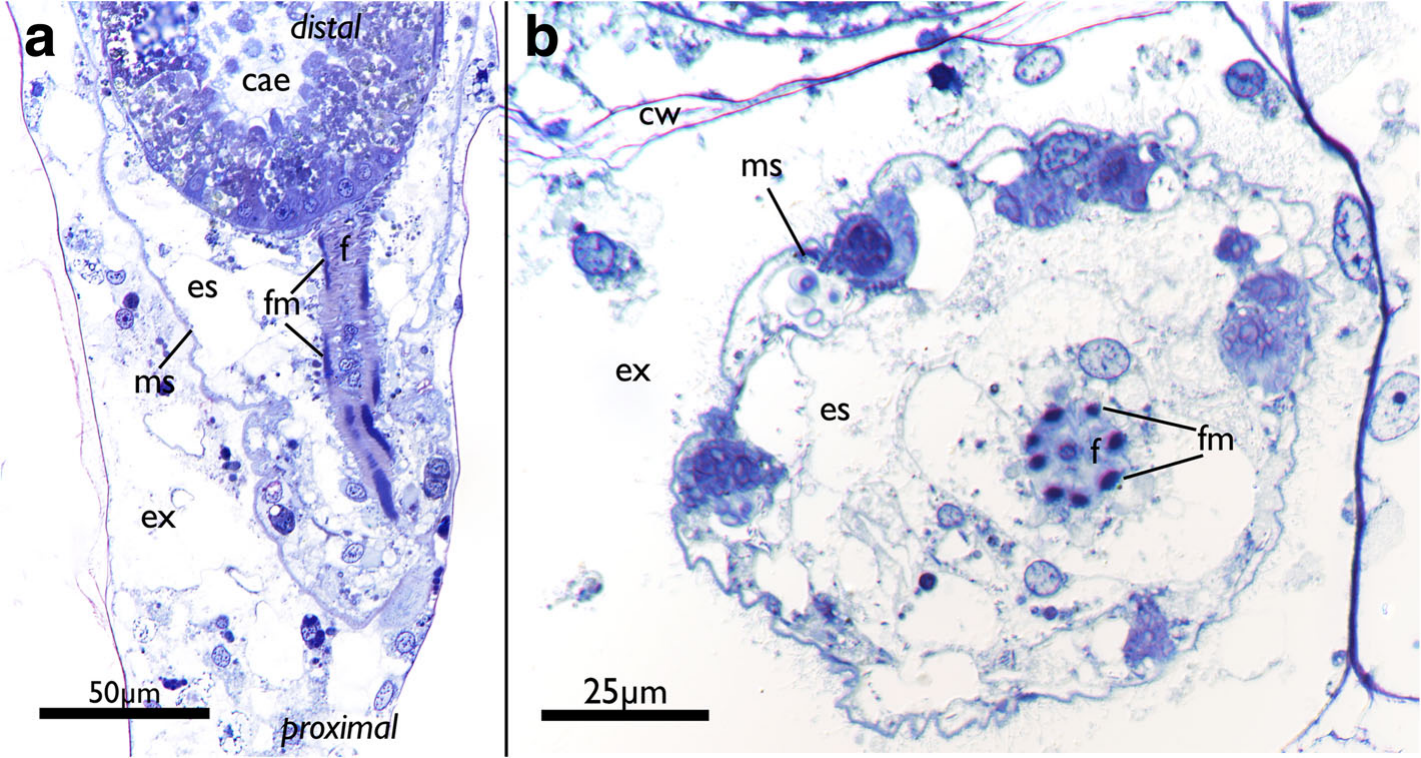
Funicular structure in *Cinctipora elegans*. Semithin sections, toluidine blue. **a** Longitudinal section of the proximal part of a zooid showing the funiculus which originates from the body wall and attaches to the caecum. **b** Cross section of a funiculus showing the regular arrangement of its longitudinal muscles. Abbreviations: cae – caecum, cw – cystid skeletal wall, es – endosaccal space (coelom), ex – exosaccal space, f – funiculus, fm – funicular musculature, ms – membranous sac (peritoneum of body wall)

*Cinctipora elegans* forms upright dense colonies with cylindrical branches. Each branch consists of tubular autozooids that extend from the median axis in an oblique-distal progression (Figs.1b, 2a–c). Polypides are located within calcified cystid walls. The latter possess regular perforations (communication pores) in the walls between zooids and some irregular protuberances on their surface (Figs. 2b, c and 3). The peritoneal layer is detached from the body wall and forms the membranous sac which surrounds the polypide and divides the zooidal cavity into ento- and exosaccal space (Fig. 4). It is connected to the cystid wall in three areas: 1) orally at the retractor muscles insertion, 2) proximally at the funiculus insertion, 3) distally in the area of the attachment organ (Fig. 4, see below). The latter attaches to the epidermis of the cystid wall almost along the entire perimetral range (i.e. almost the whole cross-section of the zooid) and leaves only small gaps of approximately 10 μm size between the main, proximal part of the exosaccal space and its parts distally of the attachment organ. The funiculus is a muscular cord without distinct lumen, but with cells filling its core (Fig. 5).

**Fig. 6:**
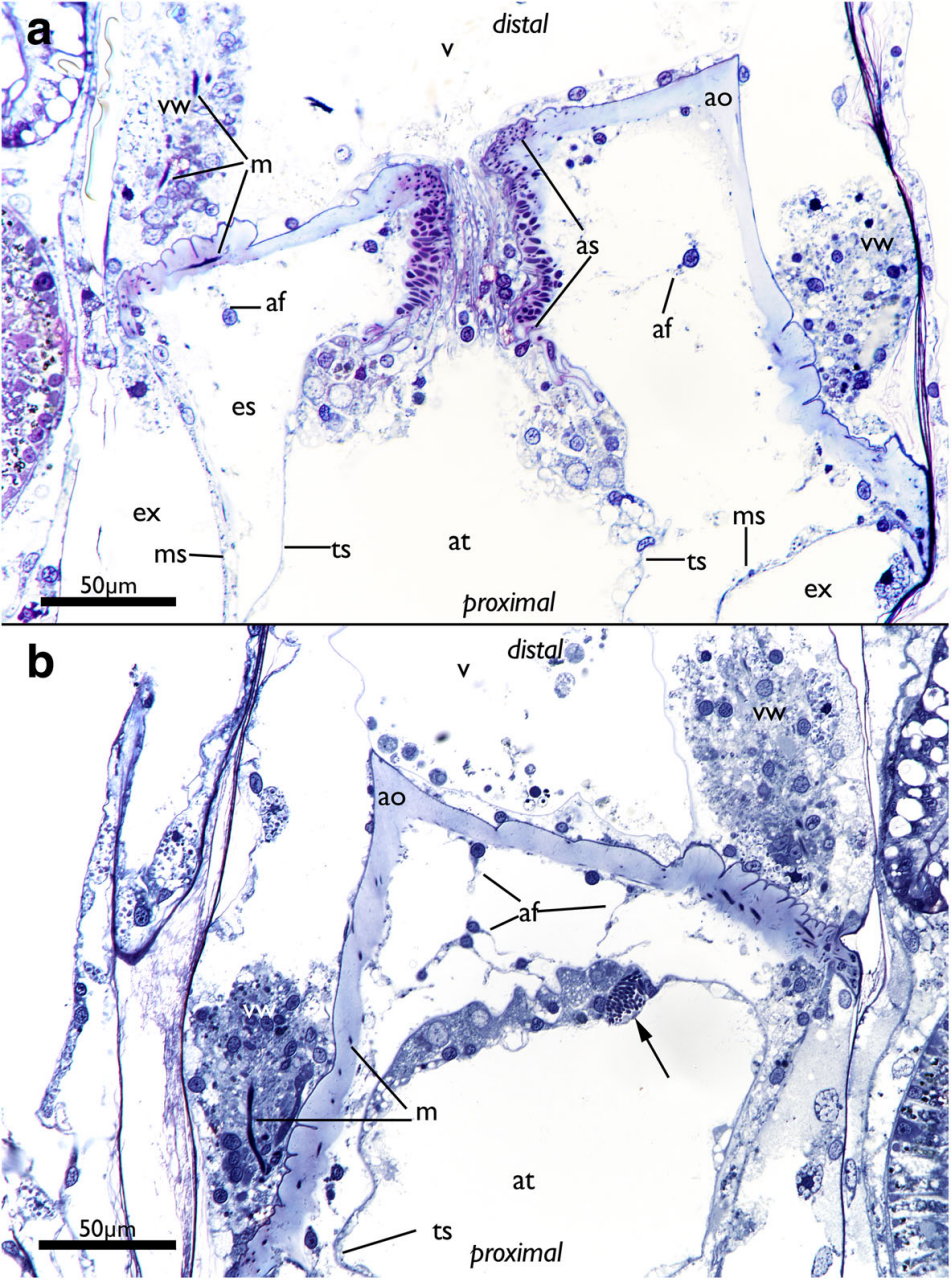
Apertural area of *Cinctipora elegans*. Histological semithin sections, toluidine blue. **a** Longitudinal section through the center of the atrial sphincter which consists of thick circular muscle fibers. The thick extracellular matrix of the attachment organ as well as the thick vestibular wall at the lateral sides of the attachment area are well visible. **b** More lateral longitudinal section showing distinct muscles in the vestibular wall and inside the extracellular matrix of the attachment organ. Note also distinct inclusions in the distal wall of the tentacle sheath (arrow). Abbreviations: af – attachment filaments, ao – attachment organ, as – atrial sphincter, at – atrium, es – endosaccal space, ex – exosaccal space, m – muscles of the vestibular wall and attachment organ, ms – membranous sac, ts – tentacle sheath, v – vestibulum, vw – vestibular wall

The attachment organ (the terminal lining of the membranous sac), lies approximately 700 μm below the skeletal aperture, which means that the lophophore can be extended only a short distance from the skeletal aperture or zooidal orifice (Fig. 2c, see also https://www.youtube.com/watch?v=2QLDnZ9Hb6M). The attachment organ consists of a very thick extracellular matrix (ECM) that proximally, towards the main body cavity, is lined by the outer epithelium of the membranous sac and distally by the vestibular wall (Fig. 6). The vestibular wall is rather thick and appears to be a multi-layered epithelium. Medially, the vestibular wall and attachment organ both terminate in the atrial sphincter (or diaphragmatic sphincter). They are also both traversed by several muscle fibers (Fig. 6).

The vestibular wall encloses a distal cavity (the vestibulum) and proximally proceeds into the atrium, which is formed by the inverted tentacle sheath in retracted polypides (Figs. 4 and 6). In such zooids, the tentacle sheath extends proximally and runs into the lophophoral base wherefrom the tentacles project distally. At the distal area of the tentacle sheath, several thin branching cells, attachment filaments, connect the proximal cellular lining of the attachment organ with the tentacle sheath. In the epithelial lining of the tentacle sheath several globular inclusions full of homogenously stained elongated bodies can be found (Fig. 6b).

**Fig. 7:**
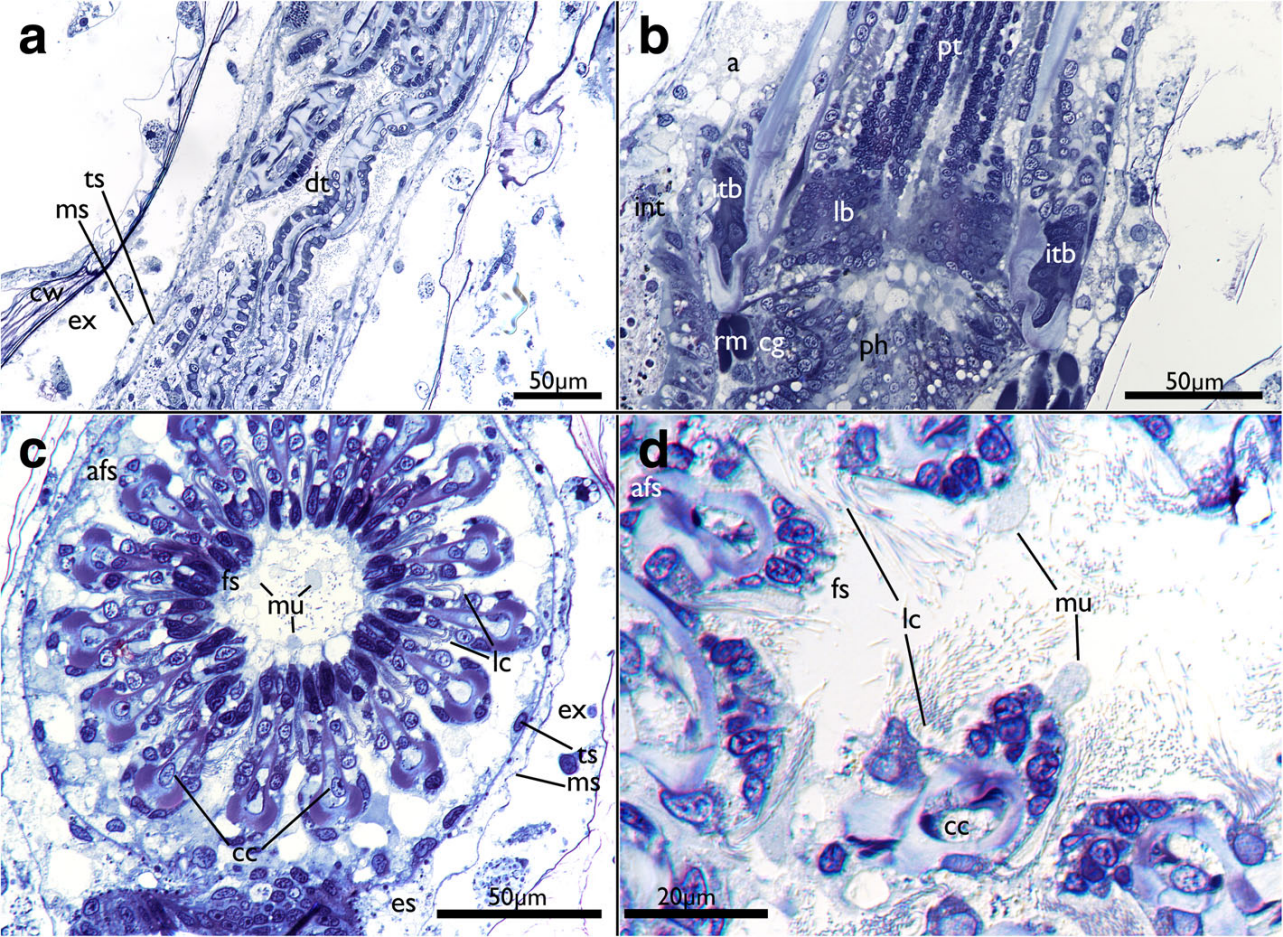
Histological details of the tentacles of *Cinctipora elegans*. Semithin sections, toluidine blue. **a** Longitudinal section of the retracted tentacle crown. The distal part of the tentacles are contorted and twisted due to contraction of the tentacle muscles. **b** Longitudinal section through the proximal part of the lophophoral base and proximal parts of the tentacles. The latter are not contorted in this area. **c** Cross-section through the proximal area of the lophophore showing the regular circular arrangement of the tentacles. Note also globular secretions, probably mucus, on the frontal side of the tentacles and enclosed within the lophophore. **d** Cross section through distal parts of the tentacles showing its more contorted form and secretory droplets being formed on their frontal side. Abbreviations: a – anus, afs – abfrontal side of tentacles, cc – central cells, cg – cerebral ganglion, cw – cystid skeletal wall, dt – distal part of tentacles, es – endosaccal space (coelom), ex – exosaccal space, fs – frontal side of tentacles, int – intestine, itb – intertentacular base, lb – lophophoral base, lc – lateral cilia, ms – membranous sac (peritoneum), mu – mucus-like secretions, ph – pharynx, pt – proximal part of tentacles, rm – retractor muscle, ts – tentacle sheath

**Fig. 8:**
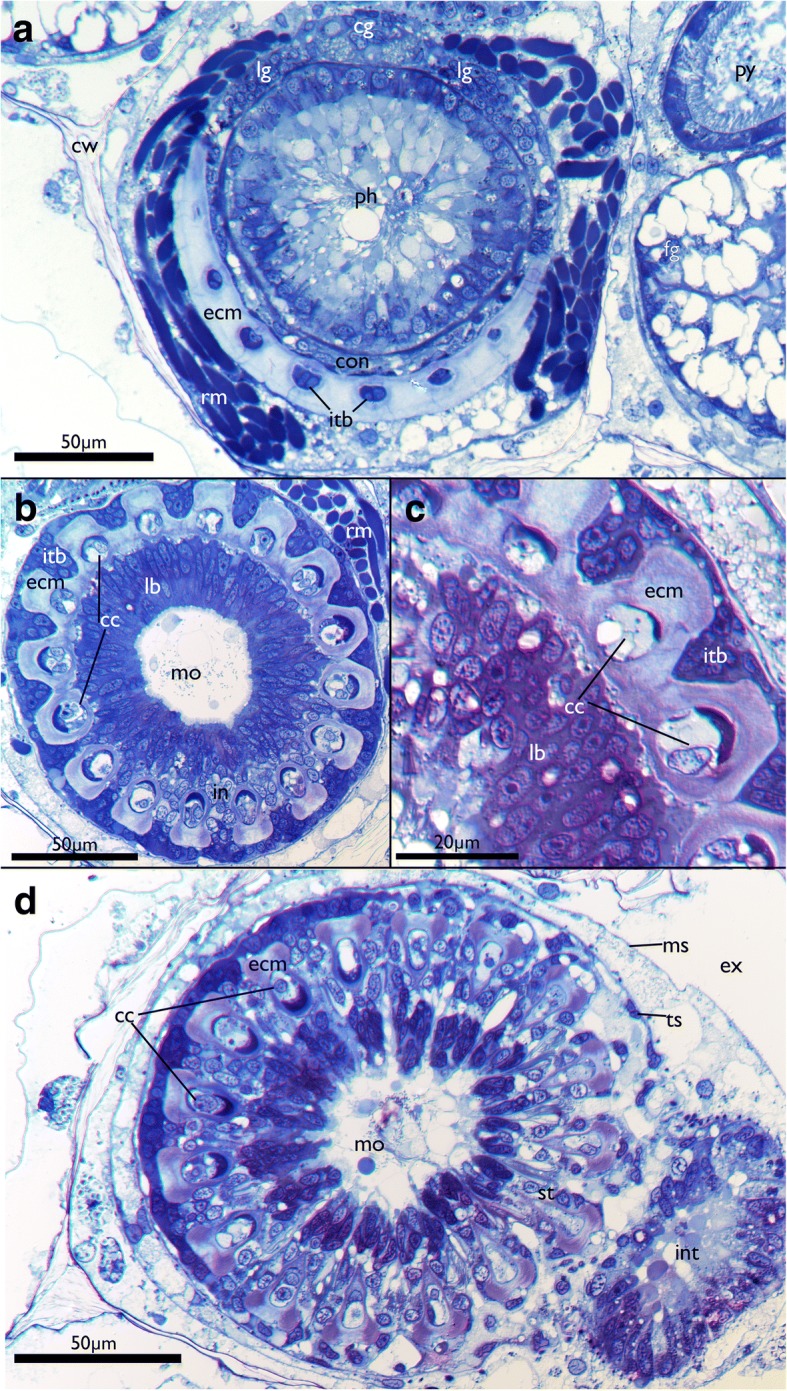
Details of the lophophoral base of *Cinctipora elegans*. Semithin cross-sections, toluidine blue. **a**, **b** and **d** are progressive sections from proximal to more distal. **a** Lophophoral base at the insertion of the retractor muscles in slightly oblique section. Directly adjacent lies the cerebral ganglion with two thick lateral ganglia and a circum-oral nerve ring encompassing the pharynx. A thick extracellular matrix and intertentacular bases are present in the proximal part of the lophophoral base. **b** Slightly more distal section than in A in the area of the mouth opening. Distinct tentacular ‘coeloms’ or cylinders are distinguishable and are surrounded laterally by the intertentacular bases. Distinct coelomic cavities are not recognizable on light microscopical level and mostly show central cells that fill the lumen. **c** Detail of the area is seen in B showing the thick extracellular matrix in the tentacle bases. **d** Most distal and slightly oblique section of the lophophoral base. Note that tentacle bases are still interconnected on the left side of the image whereas on the right they are already separated. Note also globular secretion within the area of the mouth opening. Abbreviations: cc – central cells, cg – cerebral ganglion, con – circum-oral nerve ring, cw – cystid skeletal wall, ecm – extracellular matrix, ex – exosaccal cavity, in – internal perikarya of the tentacle innervation, int – intestine, itb – intertentacular base, lb – lophophoral base, lg – lateral ganglion, mo – mouth opening, ms – membranous sac, py – pylorus, rm – retractor muscle, st – separate tentacles (distally of the lophophoral base), ts – tentacle sheath

The tentacle crown is very long and measures approx. 700 μm (Fig. 2d). However, there is only a short portion of ~ 100 μm capable of projecting outside of the zooidal aperture when polypides are protruded. At their base, the tentacles are always arranged in a perfect circle and more slender (Fig. 7b, c) whereas their more distal parts appear contorted in retracted polypides (Fig. 7a, d). Lateral cilia are readily distinguishable in histology, but the laterofrontal and frontal ones are not. The frontal side of many tentacles seems to bud off small globules of homogenously stained, acellular content (Fig. 7d). A distinct coelomic cavity could neither be observed with certainty in the lophophoral base nor in the tentacles themselves. Instead, central cells appear to fill the inner peritoneal lining of the tentacles (Figs. 7c, d and 8b-d). The tentacle ‘coeloms’/peritoneal areas or cylinders appear to emerge on the level of the circum-oral nerve ring and are embedded in a very prominent extracellular matrix at the lophophoral base (Fig. 8a-c). At the lophophoral base, the epidermal layer of the tentacles fold into intertentacular bases (Fig. 8a-c).

**Fig. 9:**
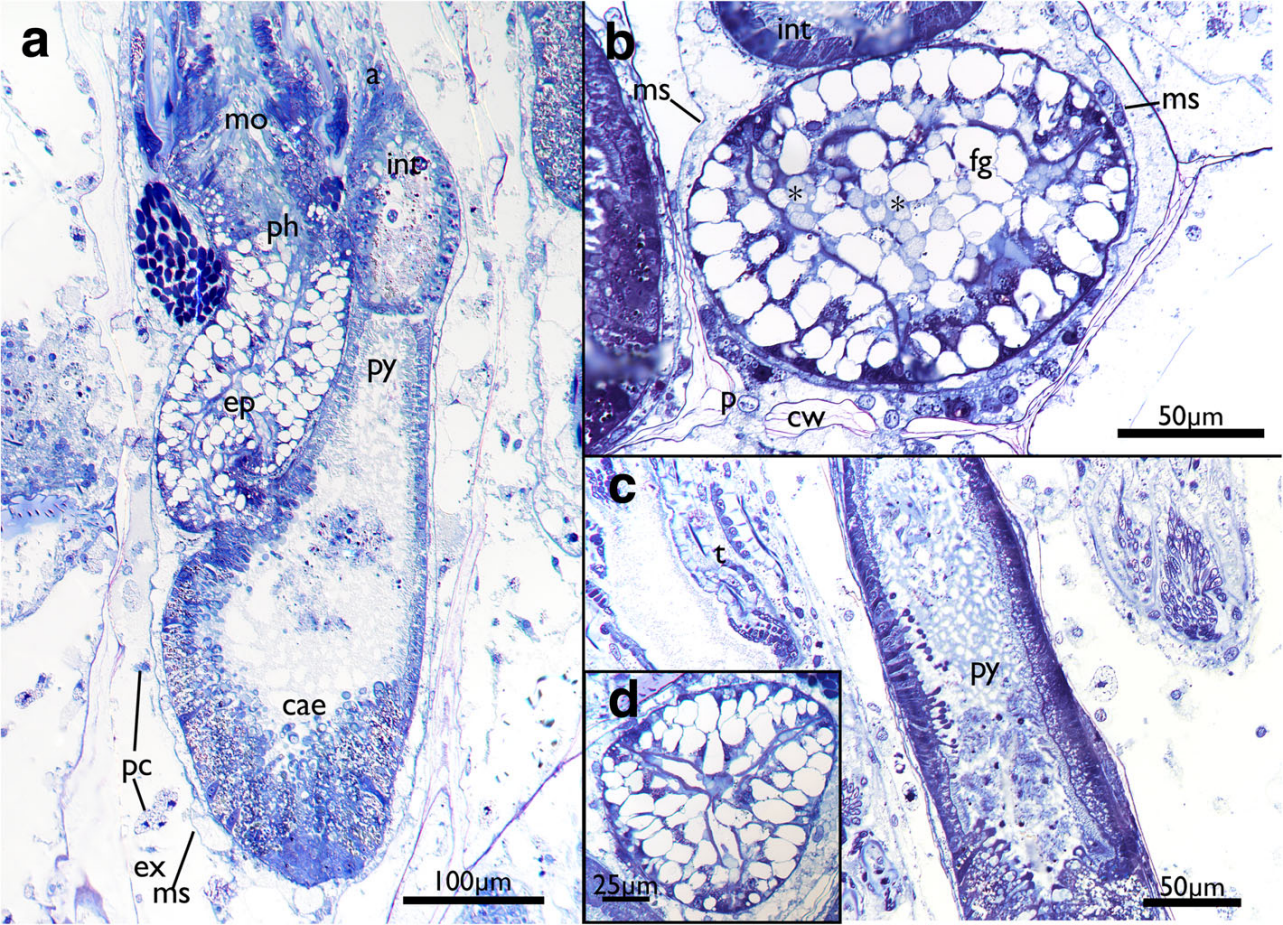
Details of the digestive tract of *Cinctipora elegans*. Semithin sections, toluidine blue. **a** Longitudinal section showing all major parts of the digestive tract. The mouth opening proceeds via the pharynx and esophagus towards the caecum. From the latter the gut continues with the ciliated pylorus before entering the intestine which terminates with the anus within the tentacle sheath. **b** Cross-section of the foregut (pharynx-esophagus) which consists of vacuolated cells. Note the lumen is filled with secretory vesicles (asterisks) as observed at the lophophoral base and the frontal side of the tentacles. **c** Longitudinal section of the pylorus showing several apical parts of the gut epithelium being shed into the gut lumen. **d** Cross-section of the pharynx showing the triradial arrangement of the lumen. Abbreviations: a – anus, cae – caecum, cw – cystid skeletal wall, ep – esophagus, ex – exosaccal cavity, fg – foregut, int – intestine, mo – mouth opening, ms – membranous sac, p – interzooidal communication pore, pc – pseudocoelomocytes, ph – pharynx, py – pylorus, t – tentacles

**Fig. 10:**
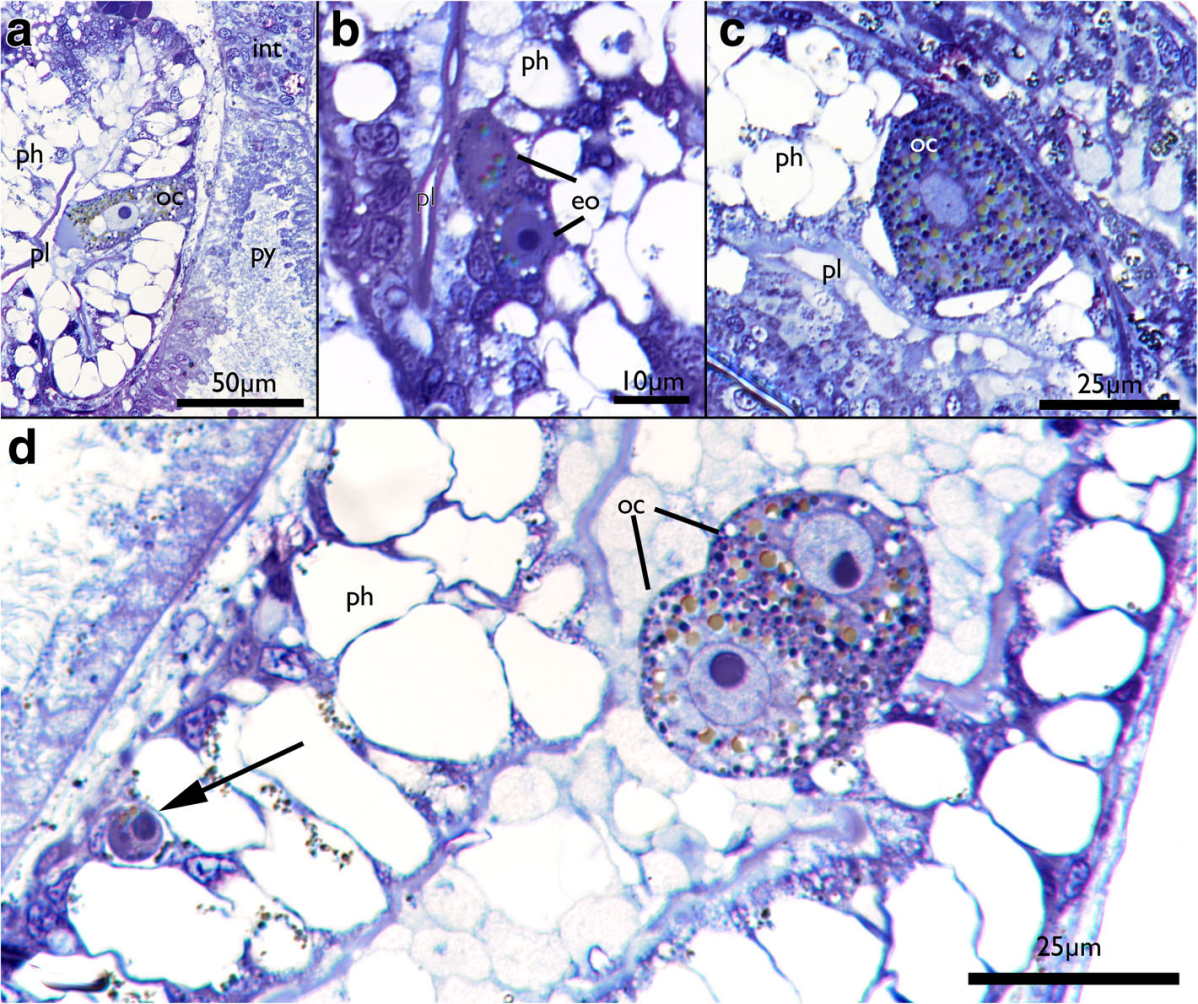
Oocytes in *Cinctipora elegans*. Semithin sections, toluidine blue. **a** Longitudinal section showing an oocyte embedded within the vacuolated pharyngeal epithelium. **b** Detail of early stages of oogenesis with two early oocytes with almost no yolk and nuclei with nucleoli occupying the largest part of the cell. **c** Detail of an oocyte in the pharyngeal wall showing the cytoplasm filled with yolk. **d** Oocyte doublet in the ‘mucus’-filled pharynx lumen. Note also the round cell (presumably oogonium) at the basal side of the pharyngeal epithelium (arrow). Also, some of the vacuolated cells of the pharynx contain granules similar to the ones in the oocytes. Abbreviations: eo – early oocyte, int – intestine, oc – oocyte, ph – pharynx, pl – pharynx lumen, py – pylorus

**Fig. 11:**
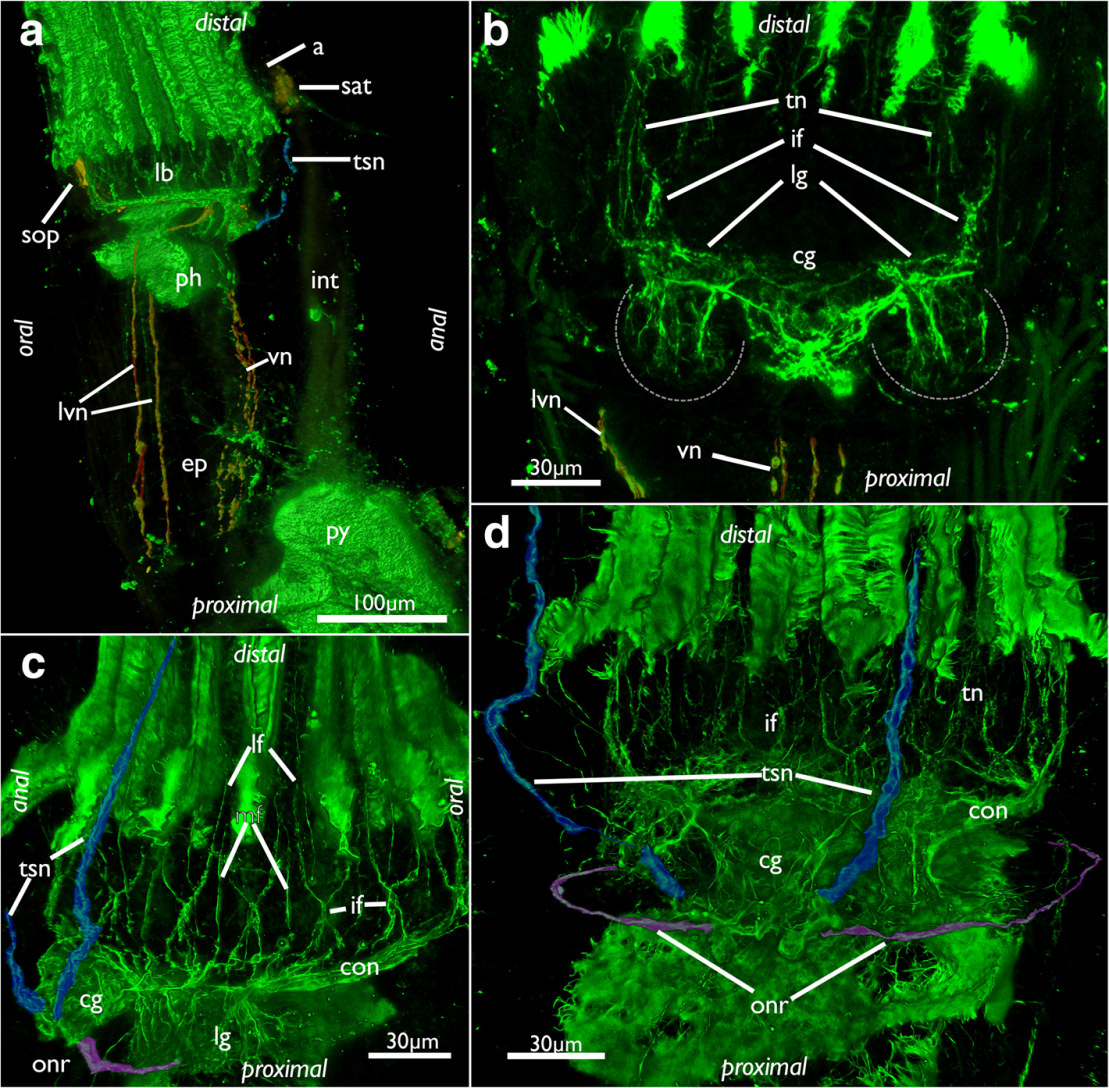
Innervation of the lophophoral base in *Cinctipora elegans*. CLSM, staining of acetylated alpha-tubulin (green) and serotonin-lir nervous system (in A, yellow). **a** Lateral view of the polypide showing the visceral neurite bundles (highlighted in red) and tentacle sheath neurite bundle (highlighted in light blue). **b** View of the anal side of the cerebral ganglion and the lateral ganglia which are proximally delineated by a dashed line. The visceral neurite bundles are highlighted in red. **c** Oblique view of the lophophoral base showing the circum-oral nerve ring, the tentacle nerves, the tentacle sheath nerves (highlighted in light blue) and the (incomplete) outer ring nerve (highlighted in purple). **d** View of the anal side showing the ganglion with the tentacle sheath nerves (highlighted in light blue) and the (incomplete) outer ring nerve (highlighted in purple). Abbreviations: a – anus, cg – cerebal ganglion, ep – esophagus, if – intertentacular fork, int – intestine, lb – lophophoral base, lf- latero-frontal nerve of tentacle, lg – lateral ganglion, lvn – laterovisceral neurite bundle, mf – medio-frontal nerve of tentacle, onr – outer nerve ring, ph – pharynx, py – pylorus, sat – serotonin-lir anal tube, sop – serotonin-lir oral perikarya, tn – tentacle nerves, tsn – tentacle sheath nerve, vn – visceral nerves

Within the lophophore base lies the mouth opening which extends into a short foregut consisting of a ciliated pharynx followed by an esophagus (Figs. 2d, 4, 7b and 9). The lumen and ciliation of the pharynx is triradiate (Fig. 9d). The lumen of the foregut is commonly filled with homogenously stained globules that are histologically identical to those found on the tentacles (Figs. 7b, 8b, 9a, b and 10a, d). The foregut extends directly into the main stomach or caecum. The cardiac valve is inconspicuous and a distinct cardia is not discernable. The caecum is a short sac that continues with the pylorus (Figs. 2d, 4, 9a, 9c and 11a). The latter is characterized by abundant cilia (Figs. 9a and c, 11a). In most parts of the lining of the mid- and hindgut, apical cell portions are elongated and seem to be secreted into the lumen where small globular elements are often observable (Fig. 9c). The most proximal epithelium of the caecum where the funiculus attaches has a different histological structure of epithelial cells devoid of any granules and seemingly little synthetic activity (Figs. 5a, 9a). The intestine follows the pylorus as a slender elongated tube terminating with the anus in the tentacle sheath above the mouth (Figs. 2a, 4, 9a and 11a).

### Oocytes

Small oocytes and oogonia were encountered wedged between cells of the pharynx in several specimens (Fig. 10a-c). Presumed spherical oogonia of about 5 μm diameter are located basiepithelially and mostly consist of a nucleus with a nucleolus surrounded by a narrow layer of cytoplasm. Oocytes are either single or in pairs, show various stages of vitellogenesis and thus contain various degrees of protein or lipid yolk inclusions. In more mature oocytes the cytoplasm is darker whereas the nucleoplasm appears lighter. Despite their small size (the largest cells were about 25 μm in diameter), the ripe oocytes can be classified as macrolecithal. Some of them also contained transparent round vacuoles of various sizes. In one instance, two apparently mature oocytes were detected within the lumen of the foregut where they were embedded in the presumable mucus-secretion (Fig. 10d).

### Nervous system

**Fig. 12:**
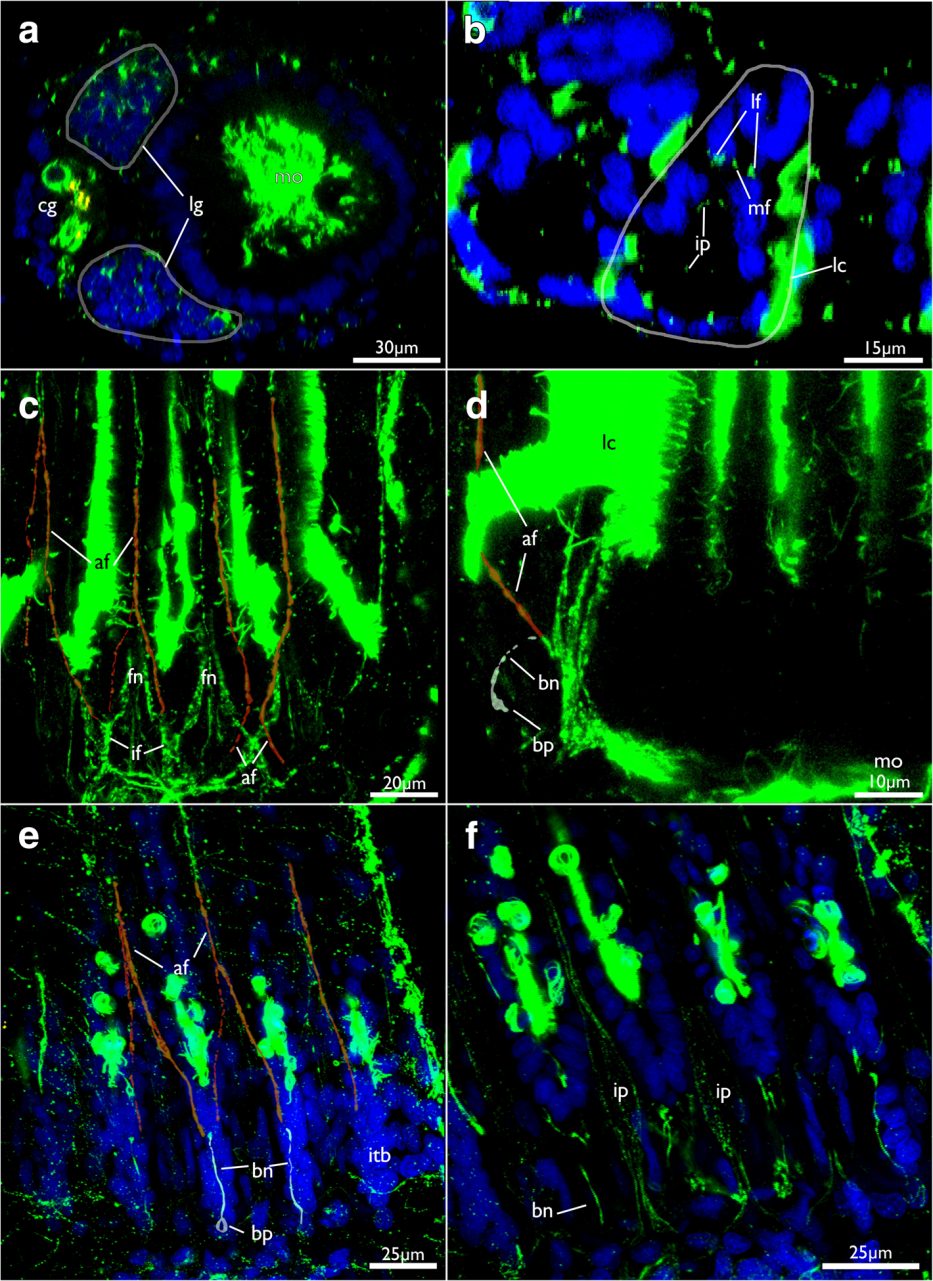
Lophophoral and tentacle innervation in *Cinctipora elegans*. CLSM, staining of acetylated alpha-tubulin (green) and cell nuclei (DAPI, blue). Abfrontal tentacle nerves are partially highlighted in red. The basal neurite bundle and perikaryon is highlighted in white. **a** Optical section of a stack through the cerebral ganglion and adjacent lateral ganglia (outlined). **b** Close-up of a virtual cross-section of tentacles (one outlined) close to the lophophoral base showing the tentacle nerves and inner peritoneal neurite bundles. **c** Maximum intensity projection image of few optical sections of the lophophoral base showing different traverses of the roots of the abfrontal tentacle nerve. **d** Projection of few optical sections showing an almost longitudinal section of an intertentacular area showing the basal neurite bundle and perikaryon at its end. **e** Optical section of the lophophoral base showing different thicknesses of the abfrontal nerve roots and the basal radial neurite bundle. **f** Optical section of the median plane of a tentacle showing distinct thin neurite bundles in the inner ‘peritoneal’ area. Abbreviations: af – abfrontal neurite bundle, bn – basal neurite bundle, bp – basal perikarya, cg – cerebral ganglion, fn – frontal nerves, if – intertentacular fork, ip – inner ‘peritoneal’ neurites, itb – intertentacular base, lc – lateral cilia, lf - latero-frontal nerve of the tentacle, lg – lateral ganglia, mf – medio-frontal nerve of the tentacle

**Fig. 13:**
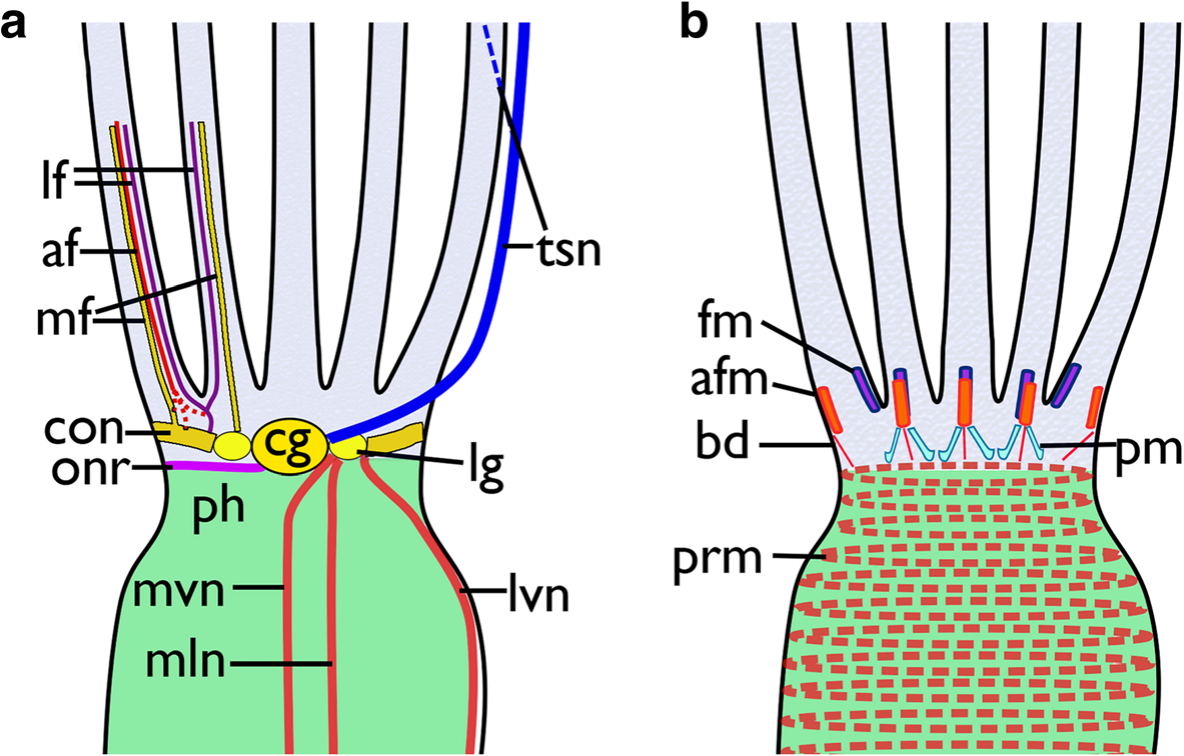
Schematic overview of the neuro-muscular system at the lophophoral base in *Cinctipora elegans*. **a** Nervous system. The tentacle innervation and outer nerve ring are only shown on the left side, and the tentacle sheath and visceral innervation on the right **b** Muscular system of the lophophoral base and pharynx. Abbreviations: af – abfrontal neurite bundle of the tentacle, afm – abfrontal muscles of the lophophoral base, bd – buccal dilatators, cg – cerebral ganglion, con – circum-oral nerve ring, fm – frontal muscles of the lophophoral base, lf - latero-frontal neurite bundle of the tentacle, lg – lateral ganglia, lvn – latero-visceral neurite bundle, mf – mediofrontal neurite bundle of the tentacle, mln – medio-lateral visceral neurite bundle, mvn – medio-visceral neurite bundle, onr – outer nerve ring, ph – pharynx, pm – proximal lophophoral base muscles, prm – pharyngeal ring muscles, tsn – tentacle sheath neurite bundle

**Fig. 14:**
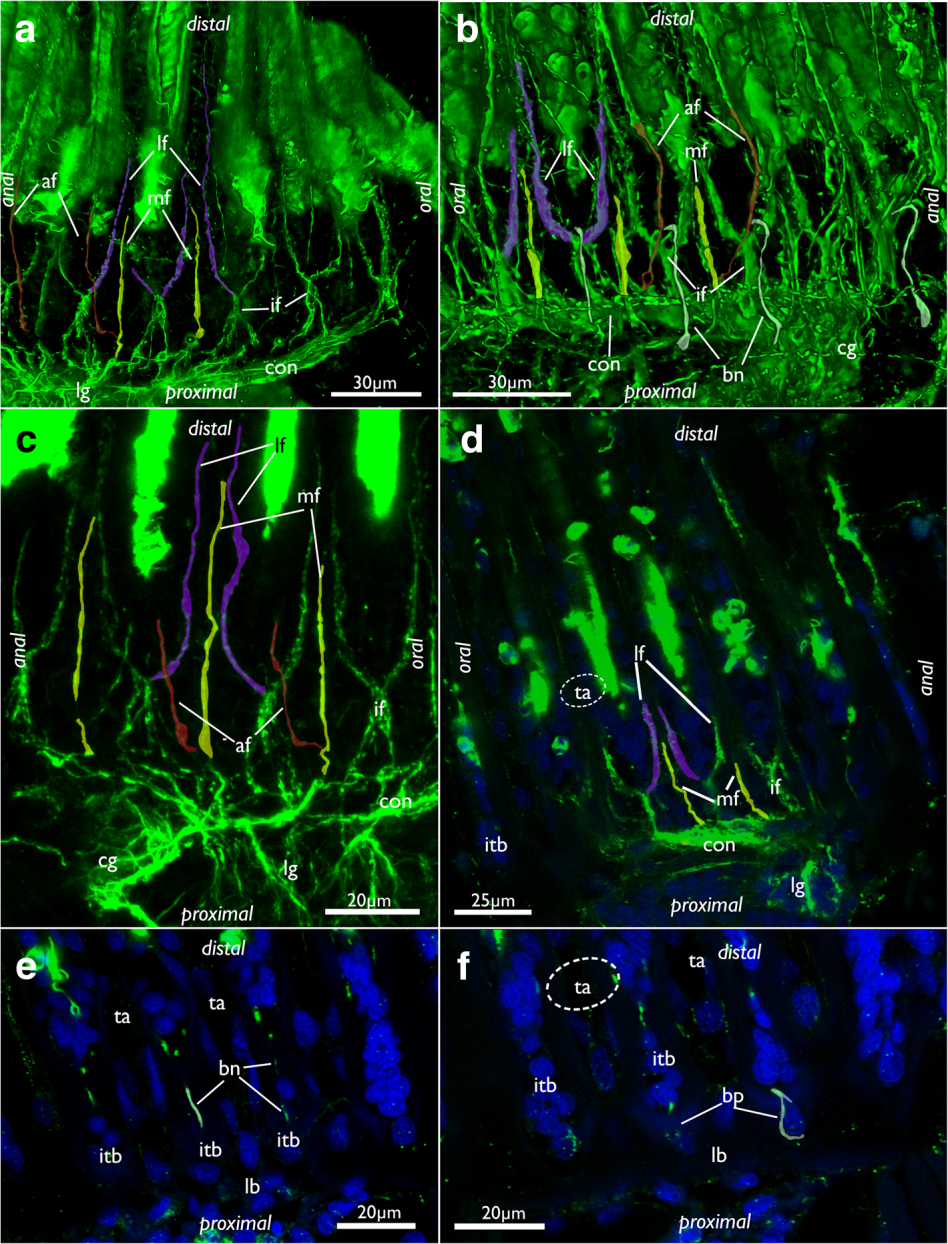
Tentacle innervation in *Cinctipora elegans*. CLSM, staining of acetylated alpha-tubulin (green) and cell nuclei (DAPI, blue). Some mediofrontal neurite bundles are highlighted in yellow, latero-frontal neurite bundles in purple and abfrontal neurite bundles in red. The basal neurite bundle is highlighted in white. **a** Lateral view of the main tentacle neurite bundles projecting from the circum-oral nerve ring. Volume rendering. **b** Similar view as in A but showing more lateral portions of the lophophore including the basal neurite bundle which originates from the intertentacular fork and extends proximally into the intertentacular base. **c** Projection images of few optical sections showing the tentacle innervation in detail. **d** Optical section of the intertentacular fork and the medio- and latero-frontal nerves. **e** Optical section of the lateral side of the lophophoral base showing basal radial neurite bundles. **f** Optical section of the same specimen as in E, but more superficially showing the basal perikarya at the proximal end of the intertentacular bases. Abbreviations: af – abfrontal neurite bundle of the tentacle, bn – basal neurite bundle, bp – basal perikarya, cg – cerebral ganglion, con – circum-oral nerve ring, if – intertentacular fork, itb – intertentacular base, lb – lophophoral base, lf - latero-frontal neurite bundle of the tentacle, lg – lateral ganglia, mf – mediofrontal neurite bundle of the tentacle, ta – area where tentacles extend distally

The cerebral ganglion is situated at the anal side of the pharynx adjacent to the intestine and close to the anus (Figs. 7b, 8a, 11b, 12a and 13a). Adjacent to the large central portion are two lateral projections in form of lateral ganglia located on each lateral side of the cerebral ganglion (Fig. 8a, 11b and 13a). The lateral ganglia are slightly smaller than the cerebral one and contain peripheral perikarya and a central neuropil (11B; 13A). The circum-oral nerve ring (CON) continues from the lateral ganglia and closes on the oral side. Several main neurite bundles emanate from the CON and innervate the tentacles (Figs. 11c, d and 14). One bundle runs directly along the longitudinal axis of each tentacle and gives rise to the medio-frontal neurite bundle (MFN) (frontal being opposed towards the mouth opening, abfrontal on the opposite, outer side of the lophophore) (Figs. 13a, 14a-d). It is not entirely clear how far the MFN extends into each tentacle: In most specimens it terminates shortly after its emergence and does not extend into the tentacles themselves. In some specimens it appears as if it fuses with one of the latero-frontal neurite bundles (see below) (Fig. 14a-c).

Other neurite bundles from the CON project intertentacularly from a single (Fig. 14b, d) or several roots that merge into the intertentacular fork (Fig. 14a, c). Shortly after its emergence from the CON, the intertentacular fork splits on the frontal side into two latero-frontal tentacle neurite bundles (LFN) in each tentacle (Figs. 13a, 14a-d). An additional short neurite bundle runs form the intertentacular fork into abfrontal direction (facing the outer side of the lophophore) (Fig. 14b, e). This basal neurite bundle extends proximally into the intertentacular bases where it terminates into a distinct perikaryon (Figs. 12d, e, f and 14b, f).

The abfrontal neurite bundle (AFN) of the tentacle has two different sites of origin: either it emerges as a thin neurite bundle from the root of the MFN from where it bends towards one of the adjacent tentacles to form the AFN root (Figs. 12c, 13a, and 14a-c). Sometimes two roots merge at the medial, abfrontal side of the tentacle to form a single AFN (Figs. 12c and 14a-c). In few cases, the AFN root also originates from the base of the MFN, directly at the CON (Fig. 14b). The second variant is that one or two abfrontal nerve roots emerge directly from the intertentacular fork where also the latero-frontal neurite bundle emanates from (Fig. 12c, e). When two abfrontal nerve roots form the AFN, one of them is distinctly thinner than the other. Also, the location where two abfrontal nerve roots merge varies (Fig. 12c, e).

Another group of very delicate tentacle neurite bundles extends from the CON into the central, ‘peritoneal’ area of each tentacle. From their emergence from the CON, they extend slightly laterally and fuse slightly more distally into a single bundle (Fig. 12b, f). These neurite bundles were not encountered in all specimens.

From each lateral side of the ganglion a pair of neurite bundles extends into the tentacle sheath. Another pair that emerges slightly proximally of the tentacle sheath neurite bundles progresses on the outer side of the lophophoral base/pharynx to form a mostly incomplete outer ring neurite bundle (Figs. 11c, and 13a). This outer ring neurite bundle runs almost in parallel direction of the CON. In addition to these two pairs of neurite bundles, several visceral neurite bundles project mainly from the lateral ganglia into the foregut (Figs. 11a, b and 13a).

**Fig. 15:**
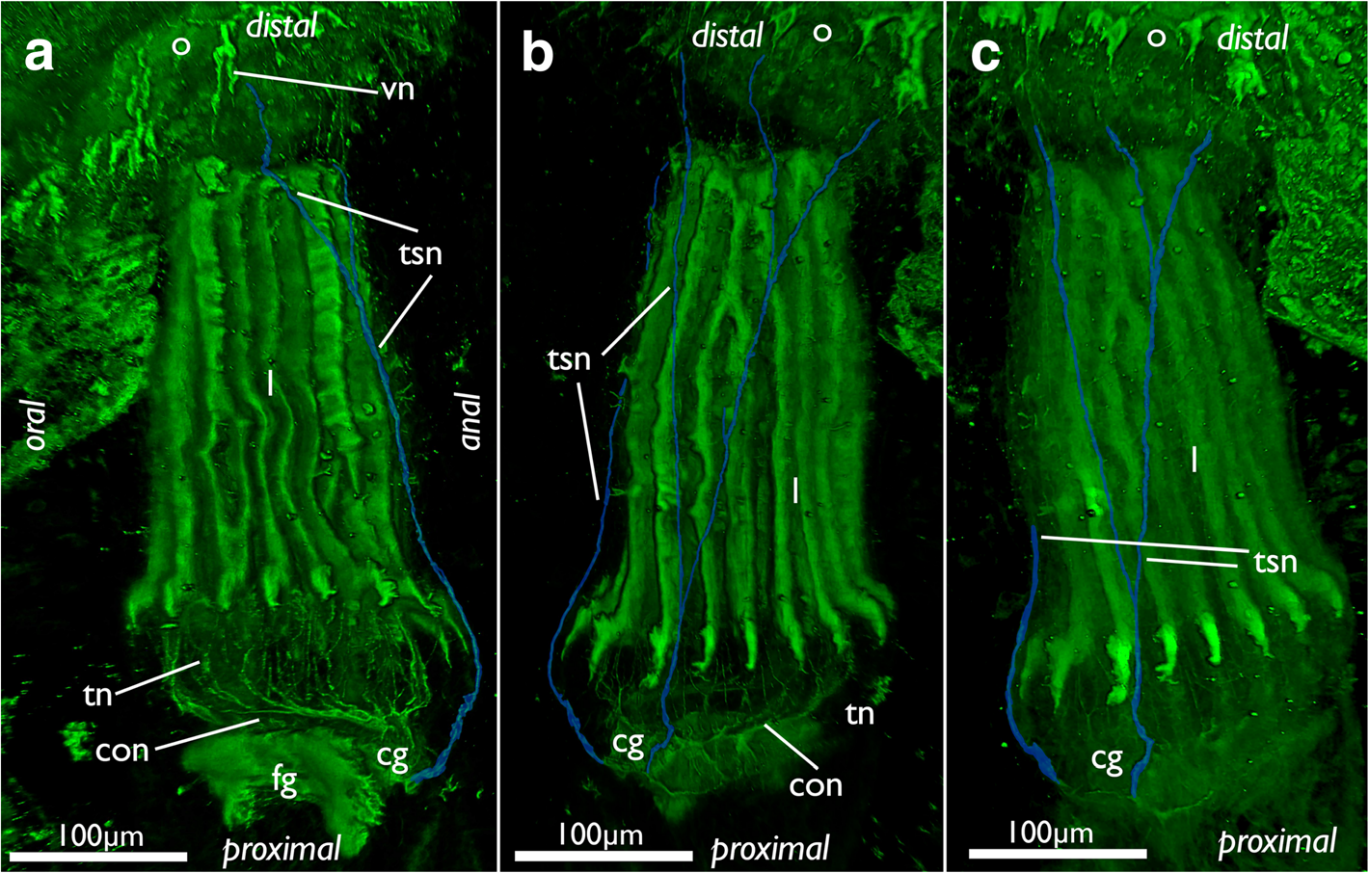
Innervation of the tentacle sheath in *Cinctipora elegans*. Stainings of acetylated alpha-tubulin (green), all volume renderings. Tentacle sheath neurite bundles highlighted in light blue. **a** Lateral view of the retracted lophophore surrounded by the tentacle sheath and its innervation on the anal side of the zooid. **b** Slight oblique view from the anal side showing the two bundles emanating from the cerebral ganglion. **c** Similar view as in B, but with more opaque rendering. Abbreviations: cg – cerebral ganglion, con – circum-oral nerve ring, fg – foregut, l – lophophore, o – orifice, tn – tentacle neurite bundles, tsn – tentacle sheath neurite bundles, vn – vestibular neurites

**Fig. 16:**
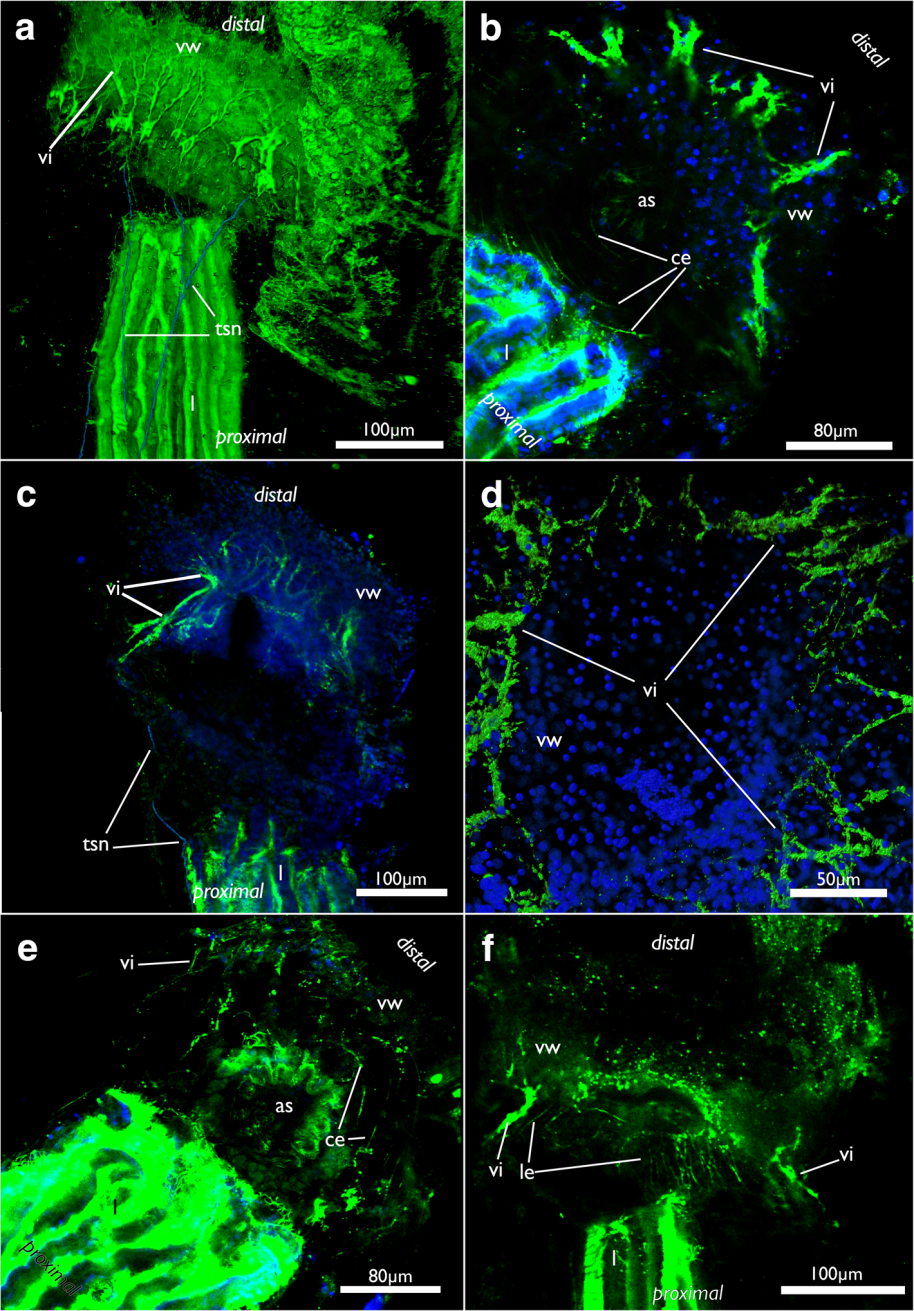
Nervous system of the apertural area of *Cinctipora elegans*, CLSM, stainings of acetylated alpha-tubulin (green) and cell nuclei (DAPI, blue). **a** Lateral view of a volume rendering of the apertural area. Tentacle sheath nerves in the proximal part are highlighted in light blue. Volume rendering. **b** Close-up showing circular neurite bundles in the extracellular matrix of the attachment organ. Volume rendering. **c** Optical section through the vestibular wall. Tentacle sheath neurite bundles in the proximal part are highlighted in light blue. **d** View from the distal side onto the apertural area showing radial arrangement of the vestibular innervation. Volume rendering. **e** Oblique view with circular neurite bundles in the extracellular matrix of the attachment organ. Volume rendering. **f** Optical section showing the thicker neurite bundles in the vestibular wall compared to the longitudinal bundles within the extracellular matrix. Abbreviations: as – atrial sphincter, ce - circular neurite bundles in the extracellular matrix of the attachment organ, l – lophophore, le - longitudinal neurite bundles in the extracellular matrix of the attachment organ, tsn – tentacle sheath nerves, vi – vestibular innervation, vw – vestibular wall

The paired tentacle sheath neurite bundles extend distally into the apertural/orificial area. Before reaching the atrial sphincter each bundle commonly splits into two fibers (sometimes twice) that further extend towards the aperture/orifice where they split into several fine neurites (Figs. 13a, 15 and 16a). The attachment organ has a regular innervation and consists of a mesh of thin radial and circular neurite bundles are located in its thick ECM (Fig. 16b, e, f). Additonal thicker neurite bundles project radially from the atrial sphincter (Fig. 16). While difficult to observe and locate, we consider the fine neurite bundles of the tentacle sheath to extend via the atrial sphincter into the attachment organ.

The visceral innervation consists in total of one mediovisceral, two medio-lateral and two laterovisceral neurite bundles (Figs. 11a, b and 13a). The former two extend from the cerebral ganglion and lateral ganglia and emanate proximally on the foregut towards the cardiac valve. The laterovisceral neurite bundles first extend on each side circum-orally to the lateral sides of the pharynx before they turn proximally to innervate the foregut until approximately to the cardiac valve as the other visceral neurite bundles do (Fig. 11a, b and 13a).

**Fig. 17:**
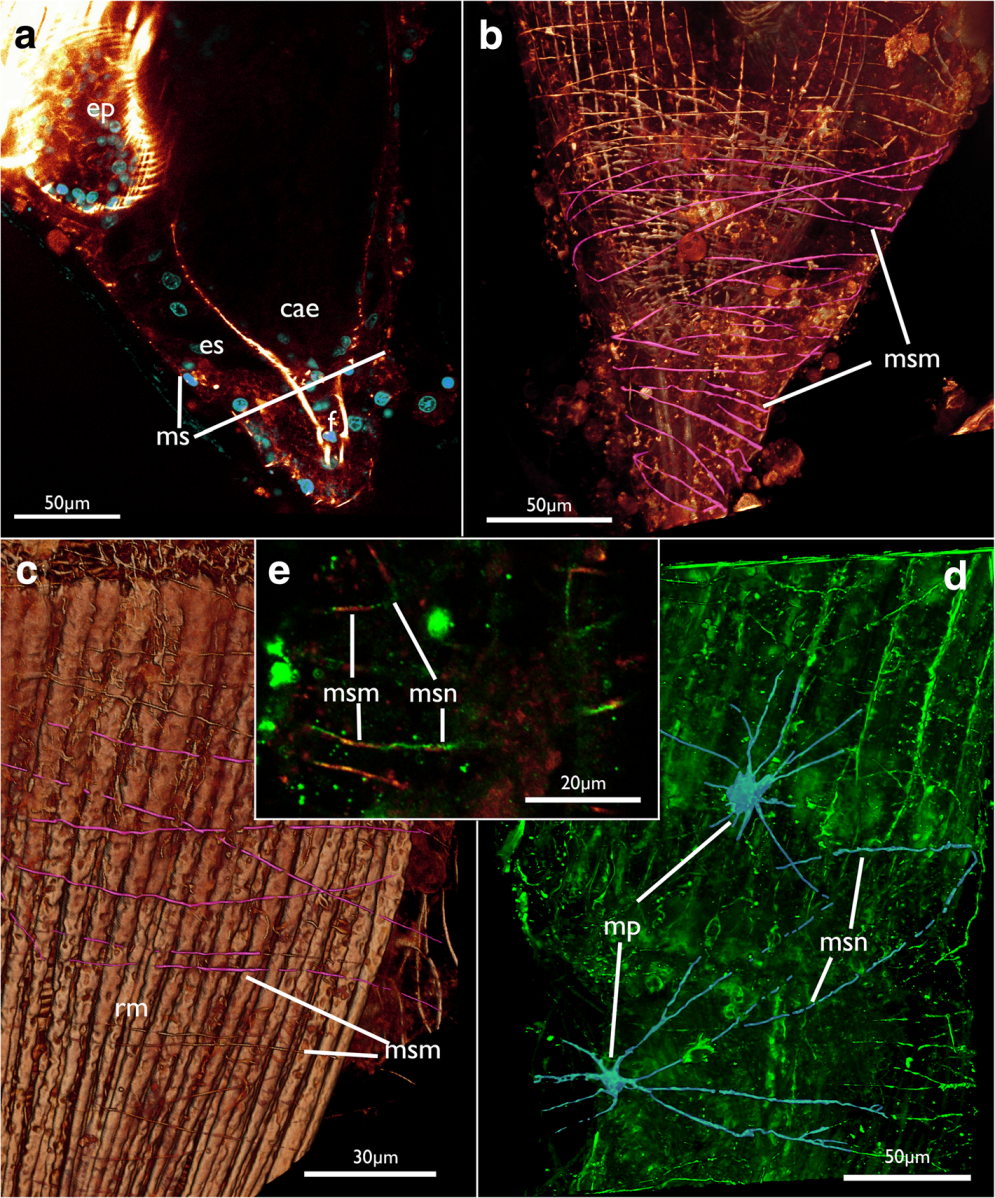
Neuromuscular system of the membranous sac in *Cinctipora elegans*. CLSM, staining of f-actin (phalloidin, glow-LUT) and acetylated alpha-tubulin (green). **a** Optical section showing the membranous sac surrounding the proximal area of the caecum. **b** Volume rendering showing the musculature of the membranous sac in the proximal area similar to A. Several fibers are highlighted in pink. Longitudinal and circular muscles of the caecum are seen beneath the membranous sac. **c** Musculature of the membranous sac in a more distal area, at the lophophoral base. Several fibers over the retractor muscle are highlighted in pink. Volume rendering. **d** Innervation of the membranous sac. Volume rendering. **e** Optical section showing muscles and nerve fibers in the same bundle. Abbreviations: cae – caecum, ep – esophagus, es – endosaccal space (coelom), mp – multipolar neurons, msm – membranous sac muscles, msn – membranous sac neurite bundles, rm – retractor muscle

The membranous sac has a rather diffuse mesh of very delicate thin neurites with predominantly circular arrangement, which reflects the situation found in the muscular system (see below; Fig. 17d, e).

### Serotonin-like immunoreactive nervous system

**Fig. 18:**
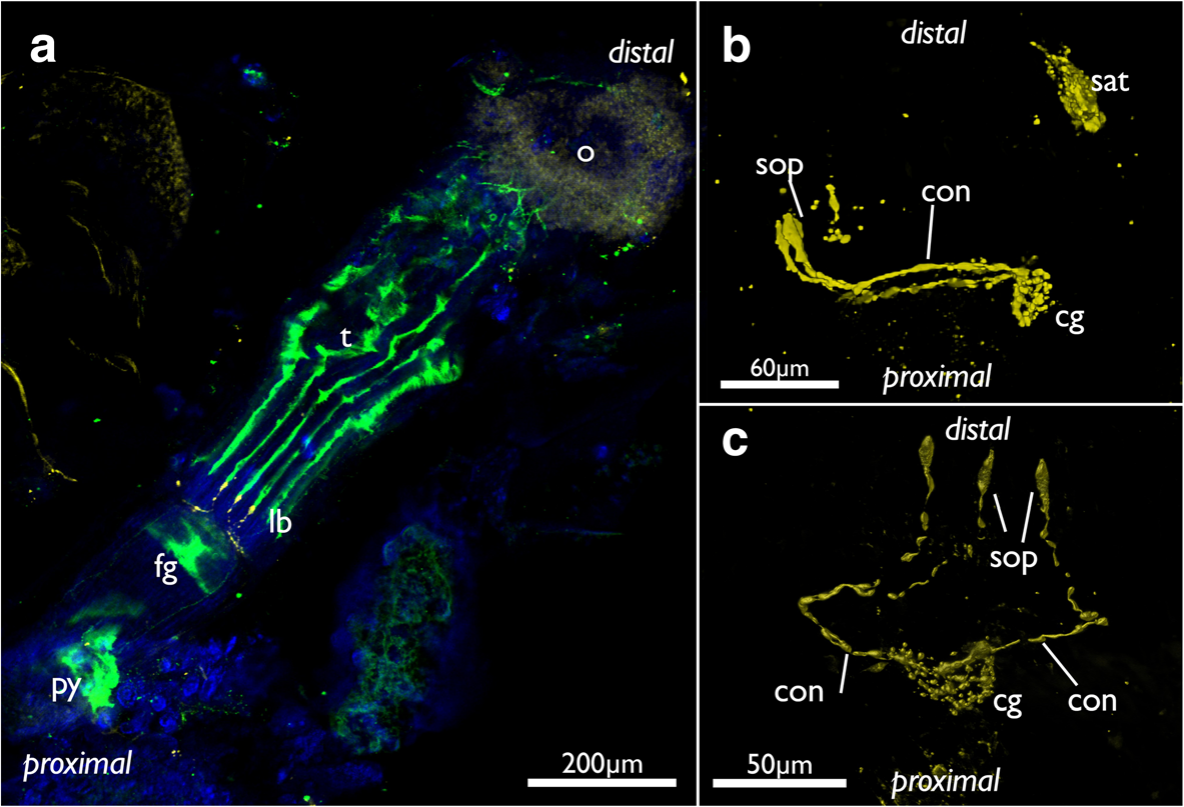
Serotonin-lir nervous system of *Cinctipora elegans*. CLSM, stainings of acetylated alpha-tubulin (green), serotonin (yellow) and cell nuclei (DAPI, blue). Volume renderings. **a** View of the oral side on a polypide showing the lophophoral base with the three oral serotonin-like perikarya. **b** Isolated serotonin-lir nervous sytem at the lophophoral base showing the cerebral ganglion, circum-oral nerve ring and the three serotonin-lir perikarya on the oral side. Also, note this specimen shows serotonin-positive staining in the anus. **c** Similar as in B but viewed from the oral side showing the three oral perikarya. Abbreviations: cg – cerebral ganglion, con – circum-oral nerve ring, fg – foregut, lb – lophophoral base, o – orifice, py – pylorus, sat – serotonin-lir anal tube, sop – serotonin-lir oral perikarya, t – tentacles

This part of the nervous system is restricted to the lophophoral base. Its main distribution is located in the cerebral ganglion (Fig. 18). From there serotonin-lir neurite bundles extend into the circum-oral nerve ring which closes on the oral side. At the latter three distinct neurite bundles extend distally towards the lophophoral base into three distinct, intertentacular, serotonin-lir perikarya (Fig. 18a, c). In one instance a fourth serotonin-lir perikaryon was detected. In addition, few zooids had distinct serotonin-lir elements in the anus (Fig. 11a). Few specimens also showed serotonin-lir signal in pharyngeal cells.

### Muscular system

**Fig. 19:**
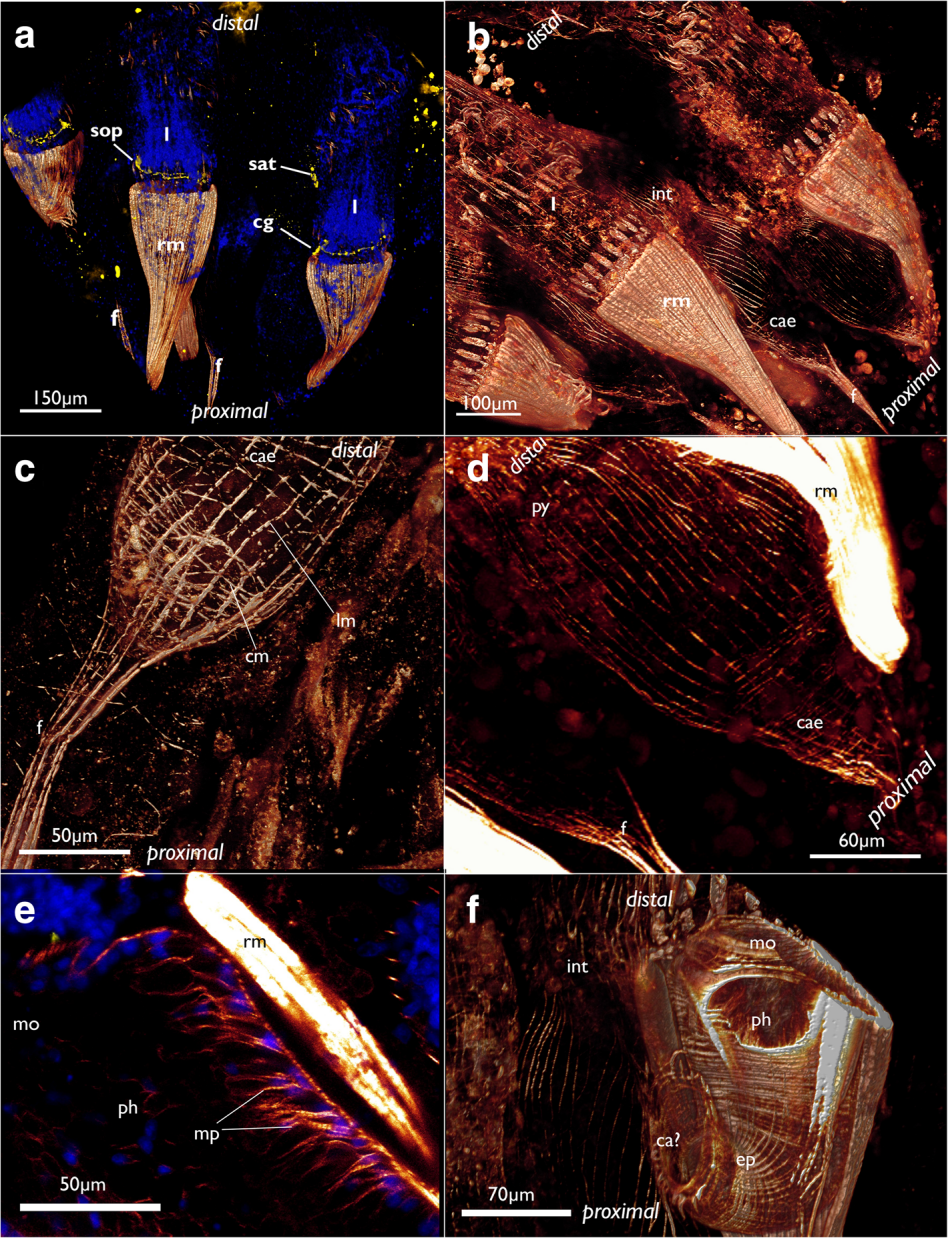
Myoanatomy of the digestive tract of *Cinctipora elegans*. CLSM, staining of f-actin (phalloidin, glow-LUT) and cell nuclei (DAPI, blue). **a** Overview of several polypides showing the prominent retractor muscles that cover most parts of the foregut. The serotonin-lir nervous system is displayed in yellow. Volume rendering. **b** Similar as in A with higher magnification of some zooids showing again the prominent retractor muscle covering the foregut and the loosely arranged, mostly thin, longitudinal musculature of the stomach (caecum, pylorus) and hindgut (intestine). Volume rendering. **c** Detail of the proximal end of the caecum showing few circular muscle fibers next to the more prominent longitudinal ones. The latter extend proximally into the longitudinal musculature of the funiculus. Volume rendering. **d** Overview of the stomach with the proximal caecum and the more distally located pylorus. **e** Optical section of the foregut showing the myoepithelial cells of the pharynx. **f** Detail of the foregut showing its dense circular as well as longitudinal muscle bundles in the esophagus towards the stomach. Volume rendering. Abbreviations: ca? – area corresponding the cardiac area of the stomach in other bryozoans, cae – caecum, cg – cerebral ganglion, cm – circular muscles of the caecum, ep – esophagus, int – intestine, l – lophophore, lm – longitudinal muscles of the caecum, mo – mouth opening, mp – myoepithelial cells of the pharynx, ph – pharynx, py – pylorus, rm – retractor muscle, sat – serotonin-lir anal tube, sop – serotonin-lir oral perikarya

The most prominent muscles in each zooid are the paired retractors (Figs. 2d, 3b, 4, 17c and 19a, b, f). Several groups of multiple thick muscle bundles run from the body wall towards the lophophoral base. All fibers are smooth. Apart from the funicular insertion and the attachment organ, the retractor muscle insertion site is one of the few contacts of the membranous sac to the epidermal layer.

The membranous sac is very thin and is adjacent to the tissues of the polypide which often makes the differentiation of tissues difficult and requires thorough study (Fig. 17a, b). Its thin wall contains circular and diagonal thin muscle fibers measuring a few micrometers in diameter over the entire range of the sac. Several diagonal fibers cross the circular ones (Fig. 17b, c).

**Fig. 20:**
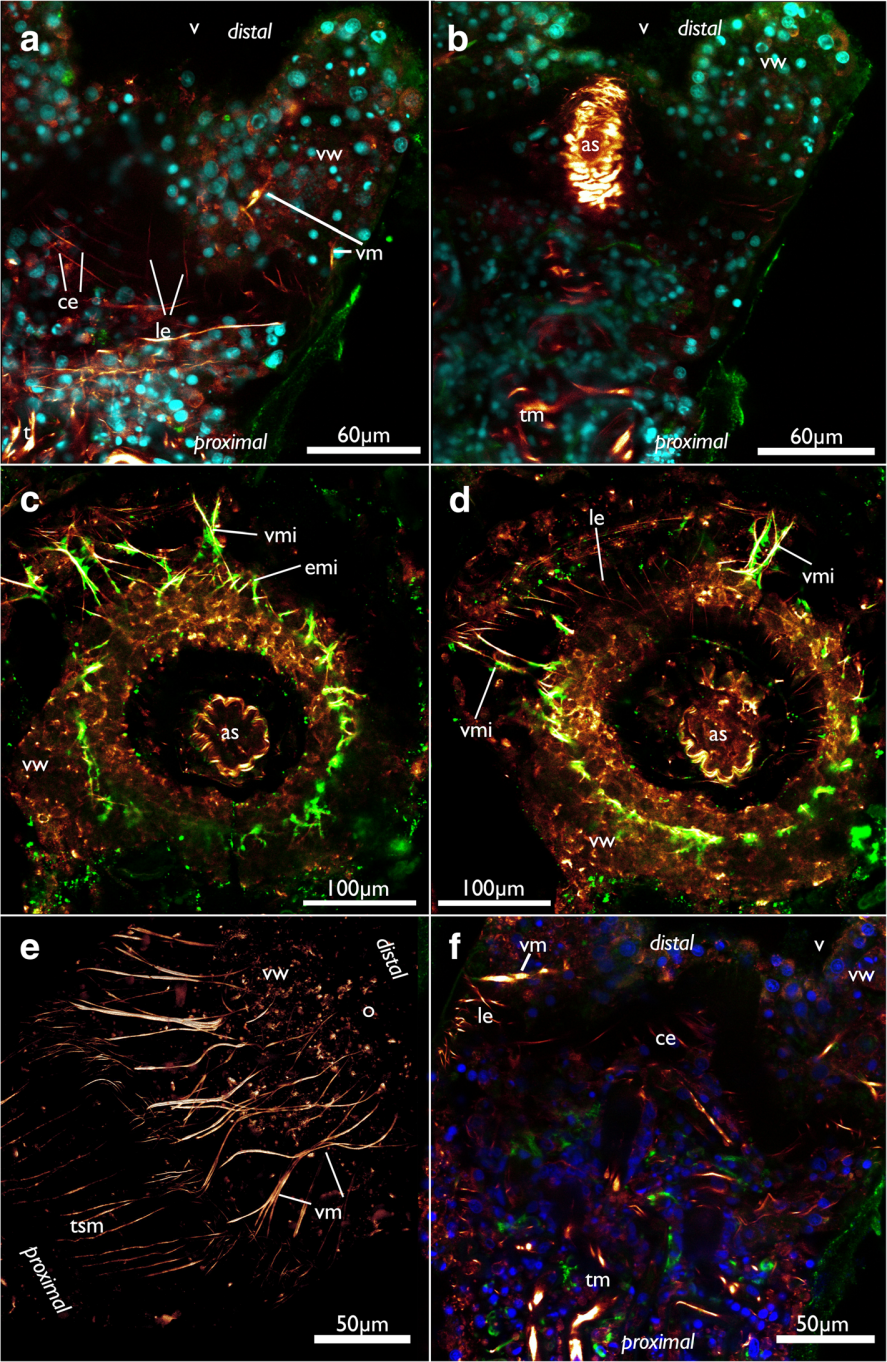
Muscular system of the apertural/orificial area of *Cinctipora elegans*, CLSM, stainings of f-actin (phalloidin, glow/red), acetylated alpha-tubulin (green) and cell nuclei (DAPI, blue or cyan). **a** Optical section through the orificial area showing the vestibular wall and longitudinal and circular muscles of the basal extracellular matrix. **b** Medial optical section of the same specimen as in A showing the atrial sphincter. **c** Optical section of the zooidal orifice. View from the distal side onto the orifice showing the rim of the vestibular wall and part of its neuro-muscular system. **d** Optical section of the same specimen as in C but located more proximally showing the thinner muscular system in the extracellular matrix underneath the vestibular wall. **e** Volume rendering of the orificial area, view from lateral side showing the arrangement of the vestibular muscles. **f** Optical section of the orificial area showing thick vestibular wall muscles vs. thinner muscles of the extracellular matrix. Abbreviations: as – atrial sphincter, ce – circular muscles of the extracellular matrix of the attachment organ, emi – muscles and neurite bundles of the extracellular matrix of the attachment organ, le - longitudinal muscles of the extracellular matrix of the attachment organ, o – orifice, t – tentacles, tm – tentacle muscles, tsm – tentacle sheath musculature, v – vestibulum, vm – vestibular muscles, vmi – vestibular muscles and neurite bundles, vw – vestibular wall

Similar to the nervous system, two sets of muscles are present in the orificial area of *C. elegans* that appear mostly co-localized with the neuronal components (Fig. 20c, d). As in the nervous system, one of the sets is located within the ECM of the attachment organ (Figs. 6 and 20a, c, d, f), whereas the other, predominantly radial muscles, is embedded in the vestibular wall (Figs. 6 and 20). The fibers in the vestibular wall are thicker than the ones in the ECM of the attachment organ. Both sets extend in the whole orificial area from the lateral body wall towards the distal tip of the atrial sphincter. The latter is a prominent tube of circular sphincter muscles that enable closure of the entrance of the atrium (Figs. 6a and 20b-d).

**Fig. 21:**
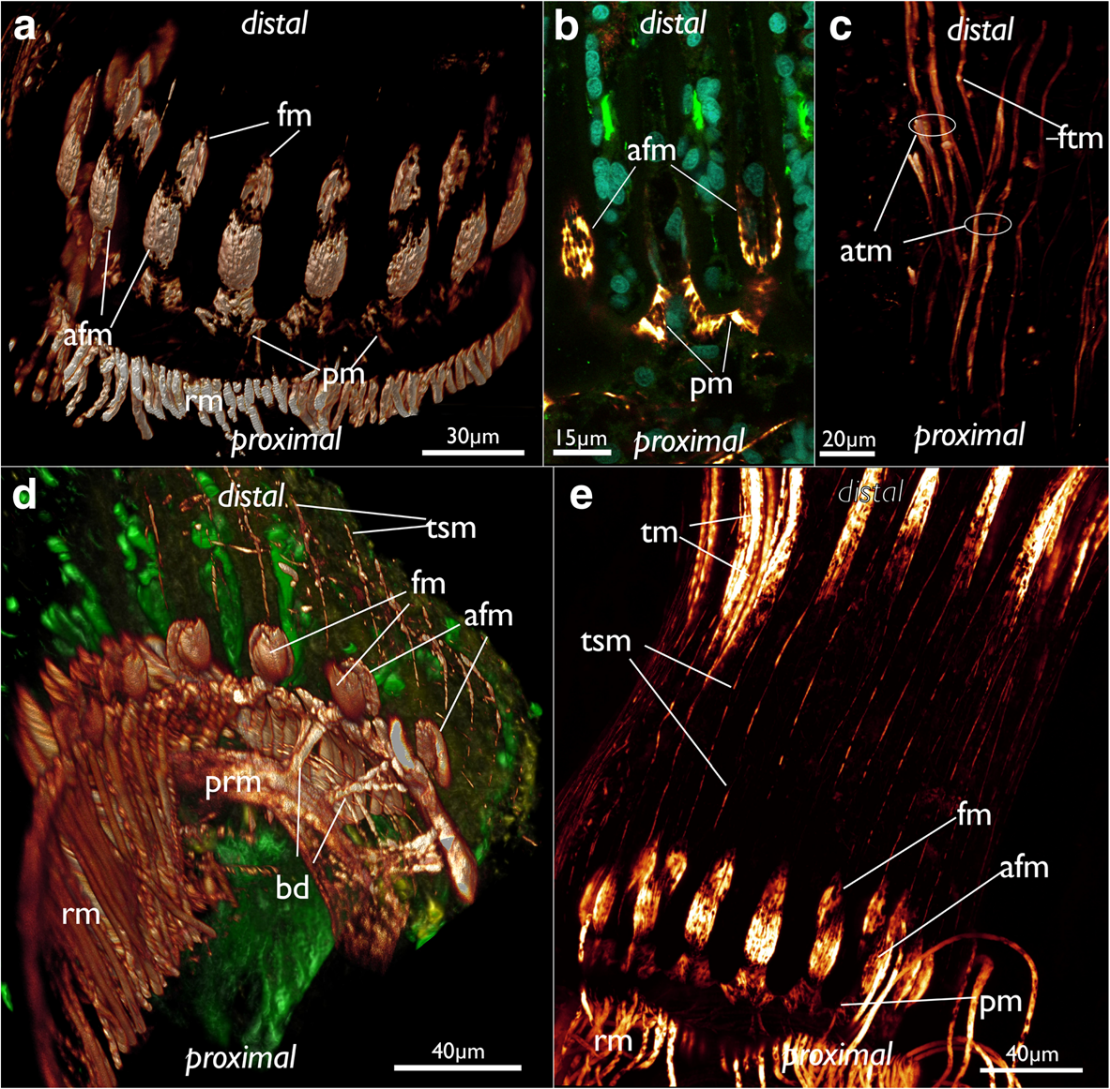
Musculature of the lophophoral base and the lophophore in *Cinctipora elegans*. CLSM, staining of f-actin (phalloidin, glow-LUT), acetylated alpha-tubulin (green) and cell nuclei (DAPI, cyan). **a** Oblique view of the lophophoral base showing the lophophoral base muscles. Volume rendering. **b** Optical section through the lophophoral base musculature. **c** Projection image of several optical sections of the tentacle muscles. Note that two separate bundles may occur on the abfrontal side. **d** Oblique view of the mouth opening and the lophophoral base showing the short buccal dilatators. Volume rendering. **e** Lateral view of the lophophoral base including tentacle sheath muscles. Note also the gap from the lophophoral base muscles until the tentacle muscles appear. Abbreviations: afm – abfrontal muscles of the lophophoral base, atm - abfrontal tentacle muscle, bd – buccal dilatators, fm – frontal muscles of the lophophoral base, ftm - frontal tentacle muscle, pm – proximal lophophoral base muscles, prm – pharyngeal ring muscles, rm – retractor muscle, tm – tentacle muscles, tsm – tentacle sheath muscles

At the lophophoral base several muscles are present (Fig. 13b): Two abfrontal muscle groups are present: one is situated more proximally and consists of two symmetrical bundles, which originate close to the pharyngeal musculature from an intertentacular position and traverse obliquely to the median plane of each tentacle. Over the whole range of the lophophoral base, this gives it almost a ‘w’-shaped appearance (Figs. 13b and 21a, b, d, e). The second abfrontal muscle is located directly above the latter. Its fibers run in proximo-distal direction (Figs. 13b, and 21a, b, d, e). On the opposite, frontal, side of the tentacle base, a single frontal muscle bundle is present. It is located slightly more distally compared to the abfrontal one (Fig. 13b and 21a, d, e). The buccal dilatators are the fourth set of muscle at the lophophoral base. These originate from the pharyngeal wall muscles obliquely in distal direction towards the abfrontal side of the lophophoral base (Figs. 13b and 21d).

A large space, devoid of musculature, follows in a distal direction from the lophophoral base into the tentacles (Fig. 21e). After this gap, tentacle muscles emerge in each tentacle. This gap is also obvious in sectioned specimens where the proximal parts of the tentacles devoid of muscles is arranged in a perfect circle and are not contorted (Fig. 7b, c), whereas distally, where they contain muscles, they are contorted (Fig. 7a, d). Tentacle muscles consist of one frontal and one abfrontal muscle bands. In several cases there were two distinct bands of abfrontal tentacle muscles (Figs. 21c, e). All tentacle muscles consist of striated muscle fibers (Fig. 21e). The whole lophophore including the lophophoral base is, in retracted zooids, surrounded by the tentacle sheath which contains only longitudinal, smooth muscle fibers (Fig. 21d, e).

The digestive tract starts with the mouth opening at the lophophoral base which enters the pharynx, followed by a short esophagus, caecum, pylorus and intestine that terminates with an anus into the tentacle sheath. The pharyngeal epithelium is a myoepithelium with cross-striated muscle fibers embedded in each lateral cell membrane (Fig. 19e). The fibers are oriented in paralell to the lateral cell membranes and extend over the whole length of the epithelial cells. The lumen of the pharynx is triradiate (Fig. 9d) and distinctly ciliated. On the outer side, beneath the gut epithelium, a dense array of circular, cross-striated muscles is situated that surround the pharynx and the esophagus. In addition to these circular muscles, thin longitudinal smooth muscle fibers are present in the foregut (Fig. 19f).

The caecum or main part of the stomach and the following parts of the gut (the pylorus and intestine) possess for the most part only a loose net of longitudinal, smooth muscle fibers (Fig. 19b-d, f). At the proximal end of the caecum, few additional circular muscle fibers are present (Fig. 19c) and its longitudinal musculature concentrates into a denser arrangement and continues as funiculus which inserts proximally on the cystid wall (Fig. 19a-d). Here, together with the funiculus, the membranous sac attaches to the epidermis and its associated calcified cystid wall.

## Discussion

### Gross morphology

The gross morphology of the polypides of *C. elegans* closely resembles that provided by a previous study [20]. However, our multi-method approach revealed numerous additional morphological details.

Regarding the tentacles, only lateral cilia were observed in *C. elegans*. Other bryozoans normally possess additional frontal and laterofrontal cilia. The former face the mouth opening and transports food particles to it, whereas the latter are stiff and presumably sensory and act as ciliary sieve [38, 39]. The lack of frontal cilia was documented in the cyclostome *Crisia eburnea* and is considered to be widespread, if not present in all Cyclostomata [26]. Ciliary sieving via latero-frontal cilia assisted by tentacle flicks effectuated by the longitudinal tentacle musculature was considered to be the major force driving food particles towards the mouth opening in cyclostomes [26]. Laterofrontal cilia might be present in *C. elegans* but not distinguishable with light microscopy and would require electron microscopy analysis.

As described for *Crisia eburnea*, *C. elegans* seems to secrete globularly packed mucus on the frontal side of the tentacles. Since these globules are also evident at the lophophoral base and mouth opening along with similar structures in the gut, it appears that mucus entrapment of the food particles could play a vital role in the feeding process of *C. elegans* as well as in other – still insufficiently studied – cyclostomes. This could also reflect several peculiarities in the digestive tract morphology in *C. elegans*, e.g. the lack of a distinct cardia otherwise common in Bryozoa [5, 40] and the apical secretions found in the cells of the stomach epithelium. The latter was also described in some gymnolaemates [41].

As reported for *Crisia eburnea* [26], a distinct tentacle lumen was not detected in *C. elegans*. Instead, central cells fill the tentacles (also reported for *C. eburnea*). Moreover, a distinct lophophoral coelom was absent, thus confirming a previous study [20]. Previous investigations of cyclostomes reported a very small coelomic cavity at the lophophoral base in the form of a ring canal which is in open connection to the tentacle coeloms. Also, the lophophoral coelom was reported to be connected to the remaining visceral coelom (endosaccal cavity) via openings or pores at the ganglion [15, 42]. To assess whether *C. elegans* has lost its lophophoral coelom, or if potentially all cyclostomes lack such a cavity, requires additional studies on more taxa. Accordingly, it remains dubious how cyclostomes could eject their sperm, which was at least for two species documented via tentacle pores [43].

Intertentacular bases as epidermal cones between tentacles located at the lophophoral base are described here for the first time in a cyclostome. These resemble the topologically identical intertentacular pits (ITP) known from some gymnolaemates [32, 44, 45]. Both also contain perikarya at their proximal border. In contrast to the ITPs, however, the cyclostome intertentacular bases are solid and do not contain any distinguishable lumen.

The funiculus of cyclostomes has hitherto been only studied in more detail in *Crisia elongata*. In this species it contains longitudinal musculature as observed in *C. elegans* and other bryozoans. However, it has a central lumen [46] reminiscent of the simple funiculus of phylactolaemate bryozoans [5]. In *C. elegans* a distinct lumen was not detected in the funicular cord.

The orificial area in all non-cyclostome bryozoans consists of several structures: distally lies the vestibular wall which represents a shallow invagination, the vestibulum, of the body wall lined by the same cuticle. Medially, the vestibular wall continues via the diaphragmatic sphincter into the tentacle sheath which in retracted zooids surrounds the cavity covering the retracted tentacles, the atrium. Connections to the cystid wall are present as thin vestibular dilatators which extend from the medial vestibular wall to the lateral body wall, and as peritoneal bands supplied with muscle fibers which extend from the tentacle sheath to the body wall [5, 28, 47].

The situation in *C. elegans* is difficult to compare to the apertural/orifical area of other bryozoans and their homology to the above-mentioned structure remains obscure. In general, the vestibular wall of free-walled cyclostomes is a continuation of the external membranous wall that is not attached to the skeletal apertural rim as previously described for *C. elegans* [20]. The vestibular wall observed in the current study consists of a thin cuticle and thick rim bordering the attachment organ and only sparse cells which sometimes form a single layer at the distal vestibular cuticle. The thickened vestibular rim appears like a folded epithelium in *C. elegans*, but confirmation requires transmission electron microscopy. Attachment organ morphology is quite diverse among cyclostomes and varies from single, asymmetrical, radially to bilaterally arranged wider attachment structures that may be lacking in some species [24, 25, 48]. All these variants allow fluid exchange from the exosaccal area towards the distal space above the attachment organ. Cinctiporids such as *C. elegans* possess an almost complete, perimetrical attachment organ with only very small gaps evident for fluid exchange from the exosaccal to the distal cavity [20, 48]. The attachment organ was previously considered to correspond to the peritoneal bands that connect the tentacle sheath with the cystid wall. Short attachment filaments that connect the tentacle sheath with the attachment organ were previously reported in *C. elegans* [20] and other cyclostomes [25, 40]. So-called ectodermal longitudinal muscles [18] situated distally of the attachment organ were considered as homologous to the vestibular dilatators in non-cyclostomes [28]. Especially the situation in *Crisia,* which possesses a terminal membrane and orificial sphincter, does not show significant similarity to *C. elegans*. In fact, drawing any conclusive statements on the structure and the evolution of the orificial/apertural area requires new histological and ultrastructural analyses over a broad range of cyclostome taxa. Based on the currently available data, most interpretations on the homology of structures within Cyclostomata and in comparison to other bryozoans remain speculative.

### Oocytes

Prior to this study, neither oocytes nor embryos or larvae had been described in Cinctiporidae which also lack gonozooids. However, the presence of ancestrulae (founding zooids from metamorphosed larvae) indicate that sexual development must take place [20]. At first glance, the encountered female germ cells appear as ingested food, but there are several arguments confirming them as oogonia and oocytes: 1) most of the cells found are embedded within the pharyngeal epithelium, 2) cells of different sizes have been found that contain different amount of yolk which seem to reflect different stages of oo- and vitellogenesis, 3) similar or identical morphology of the cells in several zooids, 4) food particles are generally not stored for a long period within the foregut but transported fast into the stomach where digestion takes place, 5) this fast transport is provided by the strong myoepithelial suction pharynx in *C. elegans* and other bryozoans [5] and 6) the cells have the appearance and size that are typical for oocytes described in other cyclostomes [15, 49–52].

Consequently, *C. elegans* possesses a unique and previously unrecognized mode of oogenesis with oocytes being formed within the epithelium of the foregut. Primordial germ cells are derived from endodermal cells in some metazoan phyla [53–55], but has so far been unknown in Bryozoa [56, 57].

Since there are no gonozooids in cinctiporids which possess very large zooids, it is likely that these zooidal polymorphs were lost in this group as indicated in the phylogeny of Boardman et al. [20] and larval development occurs inside very large autozooids after polypide degeneration. The small size of the ‘ovulated’ egg (25–30 μm in diameter) and the large diameter of the primary disc of the ancestrula (more than 0.5 mm in diameter; [20] indicates considerable enlargement of the embryo during incubation, suggesting placental nourishment as in all other cyclostomes [56]. Placentation could be a necessary prerequisite that triggered the evolution of cyclostome polyembryony [58]. We speculate that multiplication of embryos assisted by a placenta was not lost in Cinctiporidae and their reproductive pattern is with the exception of the lack of enlarged gonozooids similar to the rest of Cyclostomata.

### Nervous system

#### Gross morphology of the nervous system

The general architecture of the nervous system of *C. elegans* has many similarities to that of other bryozoan groups, particularly the Gymnolaemata [59]. As such, the nervous system is concentrated on the anal side of the pharynx as a cerebral ganglion. The ganglion is a solid mass as previously reported for cyclostomes [15, 42] and most gymnolaemates [59]. In contrast, all phylactolaemates possess a distinct lumen within the ganglion [28, 29, 60, 61]. Recently, two ctenostome gymnolaemates were found to contain a small lumen within their cerebral ganglion [30, 32]. However, the lumen in at least *A. gracilis* is minute and can only be detected with TEM [30]. Currently, the available data are not sufficient to support a specific scenario whether the lumen was lost in the Cyclostomata, Cheilostomata and most analysed ctenostomes or whether it was acquired independently in phylactolaemates and several ctenostomes.

From the ganglion an inner circum-oral nerve ring (CON) emanates and projects to the opposite, oral side of the pharynx. The CON is found in the Phylactolaemata [28, 29, 60–62], ctenostomes [28, 30, 32, 63, 64] and Cheilostomata [59, 65, 66]. Lateral ganglia on each side of the cerebral ganglion as encountered in *C. elegans* have so far not been described in any other species. The only other ganglia next to the cerebral ganglion are visceral ganglia that lie proximally adjacent to the cerebral ganglion at the foregut in a cheilostome bryozoan [67]. Interestingly, the main visceral neurite bundles of *C. elegans* originate mainly from the lateral ganglia, which would indicate a possible homology with the cheilostome visceral ganglia.

Besides the inner CON, a second nerve ring was encountered in *C. elegans* that is situated more proximally on the outer side, closer to the peritoneal lining, around the pharynx/lophophoral base. This ring corresponds to an outer nerve ring recently detected in the ctenostome *Amathia gracilis* [30]. Phylactolaemates lack any comparable second ring nerve [29, 68, 69]. In cheilostomes a topologically similar neurite bundle, the trifid nerve, emanates from the ganglion. This bundle does not form a complete ring around the pharynx/lophophoral base, but splits into neurite bundles that innervate the tentacle sheath, the foregut and the retractor muscles [67]. A similar neurite bundle is also present in some ctenostomes [32]. It seems that the outer ring of *A. gracilis* branches off similar neurite bundles to the tentacle sheath as well the foregut [30]. The former were properly identified, but the latter, visceral neurite bundles, are designated as medial tentacle sheath nerves [30], which according to their position they are not. Consequently, it appears that the roots of the neurite bundles that form the outer ring nerve are present in all gymnolaemates, but the ring itself is often reduced. A similar innervation pattern of at least two of the three neurites of the trifid nerve (visceral and tentacle sheath) support this notion. With the inclusion of the current data, it seems that an outer ring nerve appears to be ancestral for all Cyclostomata+Gymnolaemata. If the Phylactolaemata have lost it, it might altogether be ancestral for all Bryozoa.

#### Tentacle innervation

The innervation of the tentacles in cyclostomes has never been properly studied to date. The only available data are from tentacle cross-sections of *Crisia eburnea* [26] which revealed four distinct tentacle neurite bundles: three on the frontal side – one medio-frontal and two latero-frontal neurite bundles – and a single abfrontal neurite bundle. These four distinct neurite bundles are confirmed in the current study and are in general present in all Bryozoa (Phylactolaemata: [29, 61]; ctenostomes: [30, 32, 44]; Cheilostomata: [45, 67]). Phylactolaemates possess two additional latero-abfrontal neurite bundles which are lacking in Cyclostomata+Gymnolaemata [29, 61]. In *C. elegans* the medio-frontal neurite bundle terminates soon after its emergence from the circum-oral nerve ring. This could be correlated to the reduction of the frontal cilia. However, a medio-frontal neurite bundle persists in *Crisia eburnea* which also has reduced frontal cilia. Additionally, the medio-frontal neurite bundle of *C. elegans* was found to fuse with a latero-frontal one in some cases, which indicates that at least some of its bundles could be integrated into other tentacle neurite bundles. In the ctenostome *A. gracilis*, the three frontal bundles also fuse into a single neurite bundle shortly after they extend into each tentacle [30].

The origin of the tentacle neurite bundles differs in the class-level taxa of bryozoans [59]. In phylactolaemates, all tentacle nerves originate from an intertentacular position as so-called ‘radial nerves’ that split into several bundles into the adjacent tentacles [29, 70]. In the cyclostome *C. elegans*, only the latero-frontal neurite bundles have a definite origin from an intertentacular position (intertentacular fork), which corresponds to all analysed gymnolaemates [30, 32, 44, 65, 67]. The medio-frontal neurite bundle in *C. elegans* and gymnolaemates originates directly from the circum-oral nerve ring in the median plane of each tentacle and not intertentacularly. However, the situation of the abfrontal neurite bundle is complicated. In *C. elegans* the abfrontal nerve roots have three different encountered origins, namely 1) the medio-frontal nerve root, 2) the circum-oral nerve ring, or 3) the intertentacular fork. In the latter case two abfrontal roots merge to form a single abfrontal neurite bundle, which resembles the situation found in ctenostomes and phylactolaemates where the abfrontal neurite bundles originate from an intertentacular position. Sometimes, paired abfrontal nerve roots extend laterally into each adjacent tentacle from each intertentacular fork. The two roots merge medially and form a single abfrontal nerve [32, 44]. In the ctenostome *Amathia gracilis* most tentacles are only innervated by a single abfrontal nerve root which, however, still originates from an intertentacular position [30]. The second root probably was reduced in this case. The direct origin of the abfrontal neurite bundle from the circum-oral nerve ring or from the medio-frontal nerve root in *C. elegans* resembles the situation of cheilostomes where the abfrontal and medio-frontal neurite bundle arise directly from the circum-oral ring nerve [67]. The reason for this variability of the abfrontal neurite bundle is not clear and no specific pattern of its variation could be observed in *C. elegans*. The variable neurite bundle thickness of the abfrontal nerve roots and their variable origin has also been reported for the phylactolaemate *Hyalinella punctata* [61].

‘Enclosed peritoneal cells’ or ‘subperitoneal cells’ of the tentacles were described for most major clades of bryozoans (Phylactolaemata: [71], Ctenostomata: [30, 32]; Cheilostomata: [45, 65]). Sometimes, these were regarded as inner peritoneal neurite bundles. However, recent immunocytochemical studies were not able to confirm these as neurite bundles [32]. The inner peritoneal neurite bundles in *C. elegans* are the first immunocytochemical confirmation of these neurites. This indicates that six neurite bundles are present in every tentacle – four (basi-)epidermally located and two peritoneally as suggested for the cheilostome *Cryptosula pallasiana* [45]. However, it should be noted that we did not encounter these inner peritoneal neurites in all specimens.

Intertentacular basal neurite bundles as encountered in *C. elegans* are so far only distinctly known in phylactolaemates [29, 61]. In the latter, these neurite bundles have a similar traverse and terminate in an intertentacular, proximally located perikaryon [61]. Similar neuronal elements are present as serotonin-lir perikarya (see above) in gymnolaemates [33].

#### Tentacle sheath innervation

The tentacle sheath innervation of *C. elegans* consists of two bundles projecting from the lateral sides of the cerebral ganglion towards the orifice/aperture. In most specimens studied one or two additional neurite bundles branch off in distal direction. Thus, this pattern is more similar to gymnolaemates than to phylactolaemates. The latter possess a nerve plexus that spreads over the whole tentacle sheath. This plexus originates from several prominent neurite bundles emerging from the cerebral ganglion, particularly on its anal and oral sides [29, 61]. In addition, one phylactolaemate also shows additional neurite bundles that originate from the oral side of the pharynx over the ring canal {61]. In contrast, most gymnolaemates possess a single pair of main tentacle sheath neurite bundles emanating from the cerebral ganglion into apertural direction [65, 67, 72], a condition thus similar to the pair of neurite bundles observed in *C. elegans*. However, the main tentacle sheath neurite bundles in gymnolaemates are joined by a second bundle projecting from the trifid nerve. These main and additional bundles merge into the greater tentacle sheath nerve [65]. Comparing these innervation patterns, it is only clear that all large bryozoan clades possess at least two prominent tentacle sheath neurite bundles that emanate directly from the cerebral ganglion.

In the ctenostome *Amathia gracilis*, an additional tentacle sheath ring nerve running parallel to the CON is also present which emanates from two tentacle sheath neurite bundles shortly after their emergence from the cerebral ganglion [30]. A similar tentacle sheath ring nerve was also detected in the ctenostome *Flustrellidra hispida* [33]. This additional tentacle sheath nerve ring is present only in ctenostome bryozoans.

#### Visceral innervation

The visceral innervation of *C. elegans* is represented by few bundles extending into the foregut. These are concentrated on the anal side of the pharynx, but also show few neurite bundles extending from the ganglion orally before turning proximally and emanating into the foregut. Phylactolaemates possess a peripharyngeal plexus with only two more prominent longitudinal bundles on the anal side [29, 61]. The innervation pattern of cyclostomes is thus more similar to gymnolaemates where about three to five neurite bundles emerge from the anal side of the cerebral ganglion to form the lateral, medio-lateral and medial visceral neurite bundles which extend to the cardiac valve [32, 59, 67]. Corresponding medio-visceral neurite bundles are also present in *C. elegans*, medio-lateral bundles are lacking. The lateral visceral bundles of this cyclostome most likely correspond to the ones of gymnolaemates. They are slightly more orally situated in *C. elegans* than in gymnolaemates.

#### Innervation of the orifice/apertural area

The innervation of the orificial area, vestibular wall and attachment organ shows several distinct radially traversing neurite bundles that run perpendicular to the orientation of the polypide within the zooid. As mentioned above, the orificial and apertural area of *C. elegans* is difficult to homologize with corresponding structures in other bryozoans. Still, regarding the nervous system, the condition found in *C. elegans* has some similarity to Phylactolaemata where radially traversing duplicature bands contain distinct neurite bundles which are extensions of the tentacle sheath neurites towards the body wall [61]. Likewise, gymnolaemates possess parieto-vaginal bands, which are homologous to the duplicature bands, and are innervated by few neurite bundles [28, 61, 67]. However, these are not arranged radially, but present as four bands.

#### Serotonin-lir nervous system

The serotonin-lir nervous system of *C. elegans* is restricted to the lophophoral base and thus it coincides with previous descriptions on other bryozoan clades [28, 29, 33]. In the latter it is commonly associated with the cerebral ganglion, the circum-oral nerve tract and several perikarya at the lophophoral base. All previously analysed species have serotonin-like perikarya over the whole range of the lophophoral base covering most tentacles emanating from it. Particularly among the Gymnolaemata, it was recently found that most species have three distinct orally situated serotonin-lir perikarya followed by a ‘serotonergic gap’ where a pair of tentacles lacks any immunopositive signal. The remaining tentacles exhibit again a regular pattern of serotonin-lir intertentacular perikarya [33]. The three orally situated perikarya in *C. elegans* suggest a distinct homology to the three orally situated serotonin-lir perikarya of gymnolaemates. Currently it appears that these specific perikarya are shared by both Cyclostomata and Gymnolaemata. All other serotonin-lir perikarya appear to be reduced in cyclostomes, however. The encountered serotonin-lir anal tube in *C. elegans* resembles the situation found in the gymnolaemate ctenostome *Paludicella articulata* in which the serotonin-lir signal has been located within the anal linings [33]. Since this staining was not encountered very frequently, it does appear to be a very reliable character.

### Muscular system

#### Lophophoral musculature

The lophophoral base of *C. elegans* is more similar to the gymnolaemate than to the phylactolaemate condition. In Phylactolaemata, there are continuous muscular bands from the lophophoral base towards the tentacle tips whereas both, *C. elegans* and gymnolaemates, possess a distinct lophophoral base musculature separated from the general tentacle musculature [28, 32, 73]. In gymnolaemates, however, distinct lophophoral base musculature is located abfrontally and not frontally [28, Schwaha pers. obs.]. Most proximally there is either a continuous muscular ring in ctenostomes [28] or topologically similar basal transversal muscles in cheilostomes [28, 45]. These could correspond to the proximal abfrontal muscles of *C. elegans.* However, these form not a distinct muscular ring in *C. elegans*, but constitute more separated u- to w-shaped structures. The abfrontal longitudinal muscle bundles at the base of each tentacle correspond to the respective bundles in gymnolaemates [28, 73]. In contrast, gymnolaemates possess additional ‘v-shaped’ muscles distally of the abfrontal located muscles which are absent in *C. elegans*. Instead, a previously unknown distal muscle on the frontal side of each tentacle is present in this species. Similar to the analysed gymnolaemates, the function of these muscles remains enigmatic [28].

The tentacle muscles themselves consist of two longitudinal muscular bands (frontal and abfrontal) similar to both, phylactolaemates and gymnolaemates [5, 26, 28, 32, 71, 73–75]. However, a distinct, large gap between the lophophoral base muscles and tentacle muscles as observed in *C. elegans* has not been reported for any bryozoan. Interestingly, the lack of the muscles at the base is also reflected in the straight vs. contorted appearance of the tentacles in retracted zooids. Also, the occasional occurrence of two abfrontal muscle bands is also unique to this cyclostome.

#### Musculature of the digestive system

In all bryozoans the musculature of the pharynx contains striated, circular muscles [5, 28, 32]. In Cyclostomata, these circular muscles were previously identified via light microscopy in various taxa [15] as well as by transmission electron microscopy in *Crisia eburnea* [27]. Additional longitudinal muscles are uncommon in Phylactolaemata [75]. However, at least few of these fibers are present in the esophagus of a gymnolaemate ctenostome [28]. This indicates that longitudinal muscles in addition to the common circular musculature was present in the last common ancestor of the Cyclostomata+Gymnolaemata.

The gymnolaemate “Cteno”- and Cheilostomata possess a myoepithelial pharynx with distinct striated myofilaments located at the lateral cell membranes [5, 27]. Among cyclostomes, this feature had so far only been properly documented and proven in *Crisia* [27]. The present study confirms it for a second species from a distant family providing evidence that this character is probably common for the whole Cyclostomata.

The caecum, pylorus and intestine have predominantly a loose net of longitudinal musculature with only few circular fibers present at the proximal end of the caecum; all muscles are smooth. This is more similar to the situation found in Gymnolaemata where the stomach has a loose net of predominantly circular muscles with few interspersed longitudinal muscle fibers [28] or sometimes a regular net of both (Schwaha, pers. obs). Despite the apparent difference with cyclostomes having more longitudinal muscles, it still remains a rather loosely arranged muscle system of smooth fibers compared to the Phylactolaemata which possess a tight array of striated, circular muscles [32, 75]. The intestine in phylactolaemates has smooth, dense, circular muscles whereas gymnolaemates have smooth longitudinal muscles [28]. The condition of cyclostomes is thus identical to gymnolaemates. This is also reflected in the general food manipulation process where phylactolaemates knead food particles through peristaltic movements in the gut and Cyclostomata+Gymnolaemata show only few spare gut movements and rotary actions of the gut cilia [40].

#### Apertural/Orificial musculature

Concerning the muscles associated with the apertural area, only the prominent atrial sphincter had been properly described before [15, 18, 20, 24, 48]. This is the only structure that is considered homologous to the diaphragmatic sphincter of other bryozoans which also separates the proximal atrium from the distal vestibulum [28]. Other described muscles in Cyclostomata are the longitudinal ectodermal muscles (“musculi extensores vestibuli” of Borg [15]) which extend from the atrial sphincter towards the terminal membrane which closes the skeletal aperture in crisiid cyclostomes. Since a terminal membrane is absent in *C. elegans*, it is not surprising that these muscles are lacking in this species.

## Conclusions

This is the first study on the neuromuscular system of a cyclostome bryozoan based on fluorescence staining and confocal microscopy. The general architecture of these systems is more similar to gymnolaemates than to phylactolaemates (Tables 1 and 2), which is in accordance with general morphological similarities concerning the small circular lophophore and the digestive tract. With the exception of the apertural/orifical area, all main characteristics of both muscular and nervous system, i.e. digestive, lophophoral, tentacle sheath, etc. are similar to gymnolaemates (Tables 1 and 2). This supports the topology of the latest phylogenetic tree where Cyclostomata and Gymnolaemata form sister-groups [6]. Several of the characters found in *C. elegans* require the investigation of additional cyclostome species in order to draw better conclusions on a ground pattern of cyclostomes. This particularly concerns the still not well understood apertural/orificial area of these bryozoans. In addition, the newly discovered mode of oogenesis raises many questions on the reproductive biology of this species.

**Table 1:** Comparison of main muscular systems in Bryozoa

	Phylactolaemata	Cyclostomata	Gymnolaemata	Reference
funiculus	smooth, several longitudinal fibers	smooth, several longitudinal fibers	present in some species; if present smooth, longitudinal fibers	31, 75,this study
digestive tract	almost exclusively circular and striated, only intestine smooth	myoepithelial pharynx and esophagus with dense muscles, otherwise mostly smooth dispersed fibers	myoepithelial pharynx and esophagus with dense muscles, otherwise mostly smooth dispersed fibers	28; 73, this study
lophophoral base	continuous to tentacle muscles	separate abfrontal and frontal muscles	separate abfrontal muscles	28, 31, 75, this study
tentacle muscle	two longitudinal bands, smooth or striated	two longit. bands, striated; sometimes two abfrontal bands	two longit. bands, striated	31, 73, 75, this study
tentacle sheath	circ. +/or only longitudinal musc.	longitudinal musculature	longitudinal musculature	31, 73, 75, this study
body wall	orthogonal mesh of circular + longitudinal fibers	circular to diagonal muscles in membranous sac	transverse parietal muscles	18, 28, 71, this study
apertural/orificial muscles	radially arranged	radially arranged	few bundles, often bilateral symmetry	28, 75, this study

**Table 2:** Comparison of main features of the nervous system in Bryozoa

	Phylactolaemata	Cyclostomata	Gymnolaemata	Reference
digestive tract	mostly nerve plexus	few neurite bundles, on anal and lateral sides of the foregut	few neurite bundles on anal side of the foregut	29, 32, 67, this study
lophophoral base	circum-oral nerve ring	circum-oral nerve ring plus incomplete outer ring	circum-oral nerve ring plus incomplete outer ring (trifid nerve)	29, 30, 32, this study
cerebral ganglion/brain	with cavity	without cavity, plus lateral ganglia	cavity in few ctenostomes, missing in cheilostomes	30, 32, 60, this study
tentacle innervation*	6 tentacle nerves	4 tentacle nerves	4 tentacle nerves	26, 29, 32, this study
tentacle sheath	mostly diffuse nerve plexus	two neurite bundles from cerebral ganglion	two mixed bundles	29, 67, this study
body wall	nerve plexus	mainly circular neurites in epithelium of membranous sac	separate parietal neurite bundles	29, 59, 67, this study
apertural/orificial innervation	radially via duplicature bands and vestibular wall	radially via attachment organ and vestibular wall	via few parietovaginal bands or distinct aperture	61, 67, this study
serotonergic nervous system	concentrated in cerebral ganglion, thin bundles in circum-oral nerve ring, regular perikarya at lophophoral base	concentrated in cerebral ganglion, thin bundles in circum-oral nerve ring, three oral perikarya	concentrated in cerebral ganglion, thin bundles in circum-oral nerve ring, regular perikarya at lophophoral base with three oral perikarya including ‘serotonergic gap’	31, 33, this study

*possibly peritoneal neurites or subperitoneal cells not considered
